# Effects of low-carbohydrate diets versus low-fat diets on metabolic risk factors in overweight and obese adults: A meta-analysis of randomized controlled trials

**DOI:** 10.3389/fnut.2022.935234

**Published:** 2022-08-09

**Authors:** Lifu Lei, Juan Huang, Longlong Zhang, Yuqin Hong, Suocheng Hui, Jian Yang

**Affiliations:** ^1^Department of Clinical Nutrition, The Third Affiliated Hospital of Chongqing Medical University, Chongqing, China; ^2^Research Center for Metabolic and Cardiovascular Diseases, The Third Affiliated Hospital of Chongqing Medical University, Chongqing, China; ^3^Department of Endocrinology, The Third Affiliated Hospital of Chongqing Medical University, Chongqing, China; ^4^Department of Radiology, The Third Affiliated Hospital of Chongqing Medical University, Chongqing, China

**Keywords:** low-carbohydrate diets, low-fat diets, overweight, metabolic risk factors, obesity

## Abstract

**Background and aims:**

Low-carbohydrate diets (LCD) and low-fat diets (LFD) have shown beneficial effects on the management of obesity. Epidemiological studies were conducted to compare the effects of the two diets. However, the results were not always consistent. This study aimed to conduct a meta-analysis to compare the long-term effects of LCD and LFD on metabolic risk factors and weight loss in overweight and obese adults.

**Methods:**

We performed a systematic literature search up to 30 March, 2022 in PubMed, EMBASE, and Cochrane Library. The meta-analysis compared the effects of LCD (carbohydrate intake ≤ 40%) with LFD (fat intake < 30%) on metabolic risk factors and weight loss for ≥6 months. Subgroup analyses were performed based on participant characteristics, dietary energy intake, and the proportions of carbohydrates.

**Results:**

33 studies involving a total of 3,939 participants were included. Compared with participants on LFD, participants on LCD had a greater reduction in triglycerides (–0.14 mmol/L; 95% CI, –0.18 to –0.10 mmol/L), diastolic blood pressure (–0.87 mmHg; 95% CI, –1.41 to –0.32 mmHg), weight loss (–1.33 kg; 95% CI, –1.79 to –0.87 kg), and a greater increase in high-density lipoprotein cholesterol (0.07 mmol/L; 95% CI, 0.06 to 0.09 mmol/L) in 6–23 months. However, the decrease of total cholesterol (0.14 mmol/L; 95% CI, 0.07 to 0.20 mmol/L) and low-density lipoprotein cholesterol (0.10 mmol/L; 95% CI, 0.06 to 0.14 mmol/L) was more conducive to LFD in 6–23 months. There was no difference in benefits between the two diets after 24 months. Subgroup analyses showed no significant difference in the reduction of total cholesterol, low-density lipoprotein cholesterol, and blood pressure between the two diets in participants with diabetes, hypertension, or hyperlipidemia.

**Conclusion:**

The results suggest that LCD and LFD may have specific effects on metabolic risk factors and weight loss in overweight and obese adults over 6 months. At 24 months, the effects on weight loss and improvement of metabolic risk factors were at least the same. These indicated that we might choose different diets to manage the overweight and obese subjects. However, the long-term clinical efficacy and effects of various sources of carbohydrates or fat in the two diets need to be studied in the future.

## Introduction

Obesity is associated with an increased risk of hypertension, type 2 diabetes mellitus, dyslipidemia, metabolic syndrome, etc. The higher prevalence of obesity has become a significant global public health crisis issue. In 2016, more than 1.9 billion adults aged 18 years and older were overweight; of these, over 650 million were obese (1). The prevalence of obesity and its adverse consequences results in a heavy economic burden on the individual and on families and nations, including both developed and developing countries (2). Thus, improving the efficacy in preventing and controlling obesity worldwide is expected to have a great potential to reduce health costs and improve global health (3, 4).

Dietary factors play a vital role in the control of obesity. All methods of dietary intervention for obesity are based on reduced caloric diets (5). Among them, low-fat diets (LFD), especially reduced saturated fat intake, are the most widely used, which have been suggested in the dietary instruction for weight loss by the American Heart Association Nutrition Committee (6). However, in recent years, a number of studies have shown that other diets, such as low-carbohydrate diets (LCD), also have beneficial effects on significant weight loss, as well as increased energy expenditure, improved hyperinsulinemia and glycemic control, and decreased cardiometabolic risk (7–9). Thus, LCD has attracted more and more attention to the management of obesity.

Over the past 20 years, epidemiological studies have been conducted to compare the effects between LCD and LFD on metabolic risk factors and weight loss in overweight and obese adults (10–15); however, the results were not always consistent. These make people confused because both LFD and LCD have been suggested in the different dietary guidelines (6, 16, 17). Nadia et al. compared the effects of LCD and LFD on cardiovascular risk factors in healthy people (18). But this study only focused on persons without cardiometabolic diseases such as type 2 diabetes mellitus, myocardial infarction, stroke, etc., which are often accompanied by obesity and may benefit more from the two dietary patterns. Another earlier study by Hu et al. compared the effects of LCD versus LFD on metabolic risk factors in overweight and obese persons, indicating that LCD is at least as effective as LFD at decreasing weight and improving metabolic risk factors (19). After that, many new studies on this comparison are available (13–15, 20–24). The different effects on metabolic risk factors in overweight and obese persons between carbohydrate-restricted diets and fat-restricted diets still require further elucidation. Furthermore, the results of recent meta-analyses were usually conducted by medium- and short-term trials rather than separate analyses for longer-term studies, and they did not explore the effect on different populations such as patients with hyperlipidemia, diabetes, and hypertension. Therefore, we aimed to conduct the present study to compile the current evidence from all qualified randomized controlled trials to compare the long-term effects of the two diets on metabolic risk factors and weight loss in overweight and obese subjects.

## Materials and methods

### Literature search strategy

This meta-analysis was reported based on the Preferred Reporting Items for Systematic Reviews and Meta-Analyses statement (PRISMA). To evaluate the effects of low carbohydrate diets (LCD) versus low fat diets (LFD) on metabolic risk factors and weight loss. Studies that were published on or before 30 March 2022 were selected. Keywords or medical subject-heading terms were used to screen as follows: LCD, carbohydrate-restricted diet, diet, or ketogenic combined with blood glucose or blood pressure or triglycerides or total cholesterol or high-density lipoprotein or low-density lipoprotein. First, titles and abstracts were filtered to exclude irrelevant studies. The full contents of the remaining literatures were next selected according to the pre-established criteria. Furthermore, references to the selected articles were also searched. The full search strategy is shown in the Supplementary materials.

### Selection criteria

The inclusion criteria of this meta-analysis study were as follows: (1) the design of the study was a randomized controlled trial; (2) study participants were adults (at least 18 years old); (3) the subjects had a BMI ≥ 25 kg/m^2^, including overweight and obese, or BMI ≥ 30 kg/m^2^, including only obese (if the included study was from Asia, subjects with BMI ≥ 23 kg/m^2^, or BMI ≥ 30 kg/m^2^ were regarded as overweight or obese). (4) LCD and LFD were compared; (5) the intervention period was 6 months or longer; (6) both metabolic risk factors and body weight loss were included as the outcomes. Studies were excluded when other interventions such as drugs, surgery, and compulsory planned exercise were mentioned. The carbohydrate-restricted diets were defined as a prescribed intake of carbohydrates less than 40% of the total energy intake or a distinct reference to the Atkins diet, with an intake of only 20–40 g/d of carbohydrate in the first phase or carbohydrate intake of < 20% of total energy intake (25). The LFD was defined as a prescribed fat intake of less than 30% of total energy intake (25–27).

### Data extraction and quality assessment

Two investigators (LL and JH) independently searched and screened all the potential related studies. The following information from each eligible study was extracted: (1) the basic characteristics of the included studies, including author’s name, year of publication, country of origin, duration of intervention, type of design, dietary composition, number, and rate of completion; (2) the characteristics of included persons, including sample size, gender, age, BMI, and health status such as basic diseases (diabetes, hypertension, cardiovascular diseases, and hyperlipemia); (3) the changes of metabolic risk factors compared with baseline, including triglycerides (TG), total cholesterol (TC), HDL-cholesterol (HDL-C), LDL-cholesterol (LDL-C), fasting blood glucose, systolic blood pressure (SBP), diastolic blood pressure (DBP), and body weight loss.

Two investigators independently assessed the risk of bias in the included studies using the Cochrane Collaboration’s tool (28), which contains the following criteria: (1) selection bias (random method); (2) performance bias; (3) detection bias (blind method for participants and results evaluation); (4) attrition bias (incomplete result data); (5) reporting bias (selective result reporting); and (6) other sources of bias. Studies were defined as having a high risk of bias: ≥1 item was a high risk of bias, and low risk of bias if all of the items were evaluated with a low risk of bias. The others were assessed as being at moderate risk of bias. Additionally, the quality of evidence for outcomes was evaluated using the Grading of Recommendations Assessment, Development and Evaluation (GRADE), which characterizes the evidence on the study limitations, imprecision, inconsistency, indirectness, and publication bias (29, 30).

### Statistical analysis

Weighted mean differences (WMD) from baselines were calculated for the effects of LCD versus LFD on metabolic risk factors and weight loss and then a meta-analysis was performed. The baseline and outcomes values are shown in Supplementary Table 1. The heterogeneity was assessed by measuring the inconsistency (*I*^2^ statistic) of treatment effects among the trials. If there was significant heterogeneity across studies (*I*^2^ > 50%), the random effect model was used. Otherwise, the fixed effect model was used. If data were missing or incomplete, complete cases were analyzed. It should be noted that due to the different blood lipid and blood glucose units reported in the included studies, the data in mg/dL of blood lipid were converted to mmol/L by multiplying 0.0259 of TC, 0.0258 of HDL-C, 0.0259 of LDL-C, and 0.0113 of TG. The blood glucose value in mg/dL was converted to mmol/L by dividing 18. In addition, to determine whether different intervention time has different effects, the duration of intervention was stratified into 6–11 months, 12–23 months, and 24 months.

The publication bias was judged by the funnel plot and Egger’s regression test (31). Meta-regression and subgroup analyses were used to analyze the possible sources of heterogeneity, including hypertensive status, hyperlipidemia status, diabetic status, energy intake, and proportions of carbohydrates. Furthermore, sensitivity analyses were performed to explore the different potential influences by excluding each study in turn. All statistical analyses were performed using Stata statistical software (Version 14.0; Stata Corp.).

## Results

### Results of literature search

**FIGURE 1:**
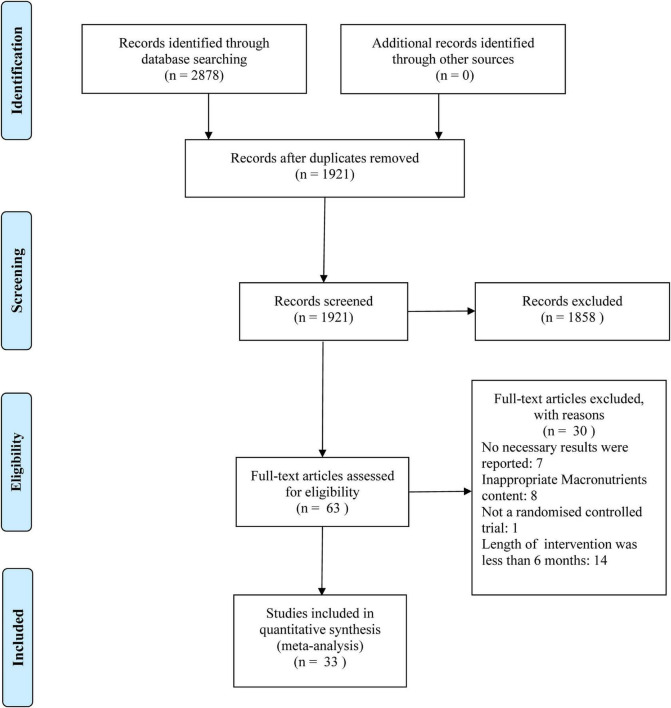
Flow diagram of study selection and meta-analysis.

The flow diagram of the study screening procedure is shown in Figure 1. A total of 2,878 potentially relevant studies were retrieved. Based on the aforementioned criteria, 1921 articles were discharged after reviewing the titles and abstracts. After evaluating the full texts, 30 of the 63 studies did not meet the inclusion criteria and were removed. Finally, 33 studies met all inclusion criteria and were selected for further analysis in this study.

### Characteristics of the included studies

The basic characteristics of 33 randomized controlled trials included in this meta-analysis are shown in Table 1 (10–15, 20–24, 32–53). Nineteen of the included studies were conducted in North America, three in Asia, four in Oceania, and seven in Europe. A total of 3,939 participants, 1,978 on LCD and 1,961 on LFD, were included. The mean age of the participants at baseline ranged from 18 to 72 years. The follow-up period ranged from 6 to 24 months, eleven in 6 to 11 months, eighteen in 12 to 23 months, and four in 24 months. All studies were conducted among overweight or obese persons with or without basic diseases such as diabetes, hypertension, and hyperlipemia.

**TABLE 1:** Characteristics of randomized controlled trials included in the meta-analysis^1^.

First author, (Reference no.)	Country	Duration of follow-up (months)	Design	No. of participants	Age (mean)	Male (%)	Population	Dietary composition		Completion %		Outcome measures
								LCD	LFD	LCD	LFD	
Bazzano, (15)	American	12	Parallel	148	46.8	12	Overweight/obese; BMI: 30–45; no T2DM or CVD	Carbohydrate intake <40 g/d, no set energy goal	<30% of daily energy intake from total fat (with <7% from saturated fatty acids), 55% carbohydrate	60 79%	59 82%	WL, SBP, DBP, TC, TG, LDL-C, HDL, BG
Brinkworth, (35)	Australia	12	Parallel	69	51.5	36	Overweight/obese; BMI = 21; metabolic syndrome risk factor	61% fat (20% saturated fatty acids), 4% carbohydrate, 35% protein	30% fat (8% saturated fatty acids), 46% carbohydrate, 24% protein	33 60%	36 69%	TC, TG, HDL-C, LDL-C, WL, SBP, DBP, BG
Brehm, (10)	American	6	Parallel	42	43.7	0	Obese; BMI: 30–35; no DM or CVD	Carbohydrate intake =20 g/d	30% fat, 55% carbohydrate, 15% protein	22 85%	20 74%	TC, TG, HDL-C, LDL-C, WL, SBP, DBP, BG
De Luis, (13)	Spain	9	Parallel	331	50.1	25.7	Obese; BMI: 35.4 ± 5.3; no DM or CVD	40% fat, 33% carbohydrate (86.1 g/day), 20% protein	27% fat, 53% carbohydrate, 20% protein	168 100%	163 100%	TG, TC, HDL-C, LDL-C, WL, SBP, DBP, BG
Davis, (37)	American	12	Parallel	105	55	21.9	Overweight/obese; BMI =25;T2DM	49% fat, 24% carbohydrate, 27% protein	25% fat, 53% carbohydrate, 22% protein	55 100%	50 100%	TC, TG, HDL-C, LDL-C, WL, SBP, DBP
Dansinger, (36)	American	12	Parallel	41	48	52.5	Overweight/obese; BMI: 27–42; at least one metabolic cardiac risk factors	Carbohydrate intake =20 g/d, and increasing up to 50 g/d	Vegetarian diet, 10% of calories from fat	21 53%	20 50%	TC, TG, HDL-C, LDL-C, WL, SBP, DBP, BG
Elhayany, (39)	Israel	12	Parallel	124	56.4	53.2	Obese; BMI: 27–34; T2DM	45% fat (50% monounsaturated fatty acid), 35% carbohydrate, 20% protein	30% fat, 50–55% carbohydrate, 15–20% protein	61 72%	63 71%	TC, TG, HDL-C, LDL-C, WL, BG
Ebbeling, (38)	American	18	Parallel	73	27.5	20.5	Obese; BMI: = 30; no DM	35% fat, 40% carbohydrate, 25% protein	20% fat,55% carbohydrate, 25% protein	28 78%	23 62%	TG, HDL-C, LDL-C, WL, SBP, DBP, BG
Foster, (50)	American	24	Parallel	307	45.5	32.2	Obese; BMI: 30–40; no DM	Carbohydrate intake 5 g/d per week	30% fat, 55% carbohydrate, 15% protein	153 58%	154 68%	TG, HDL-C, LDL-C, WL, SBP, DBP
Frisch, (41)	Germany	12	Parallel	200	47	31	Overweight/obese; BMI: = 27; no DM	>35% fat, <40% carbohydrate, 25% protein	<30% fat, >55% carbohydrate, <15% protein	100 85%	100 80%	TC, TG, HDL-C, LDL-C, WL, SBP, DBP, BG
Foster, (40)	American	6	Parallel	63	31.7	47	Overweight/obese; BMI: =21; no DM	Carbohydrate intake <20 g/d	25% fat, 60% carbohydrate, 15% protein	33 61%	30 57%	TC, TG, HDL-C, LDL-C, WL, SBP, DBP
Gardner, (23)	American	12	Parallel	609	40	43	Overweight/obese; BMI: 28–40; metabolic syndrome	The mean 12-month macronutrient distributions: 45% fat, 30% carbohydrate, 23% protein	The mean 12-month macronutrient distributions: 29% fat, 48% carbohydrate, 21% protein	218 74%	214 74%	TG, HDL-C, LDL-C, WL, SBP, DBP, BG
Guldbrand, (51)	Sweden	24	Parallel	61	62	44.3	Overweight/obese; BMI: 32; T2DM	50% fat, 20% carbohydrate, 30% protein	30% fat (<10 % saturated fatty acids), 55–60% carbohydrate, 10-15% protein	30 100%	31 100%	SBP, DBP, BG, LDL-C, HDL-C, TG
Gardner, (42)	American	12	Parallel	153	42	0	Overweight/obese; BMI: 32; no DM	Carbohydrate intake =50 g/d	<30% of total energy intake from fat	68 88%	59 78%	TG, HDL-C, LDL-C, WL, SBP, DBP, BG
Haufe, (20)	American	6	Parallel	170	44	20.6	Overweight/obese; BMI: 26.5–45.4, no T2DM	Carbohydrate intake =90 g/d	Fat intake of =20% of total energy	55 66%	56 64%	WL, TG, LDL-C, HDL-C, BG, TC
Hockaday, (43)	UK	12	Parallel	93	51	55.9	Weight: 76.4–82.2 kg	40% fat, 20% carbohydrate, 20% protein	26% fat, 54% carbohydrate, 20% protein	54 NR	39 NR	TG, BG
Jonasson, (14)	Sweden	6	Parallel	61	62	44.2	Overweight/obese; BMI: 33; DM	43% fat, 20% carbohydrate, 31% protein	30% fat, 55–60% carbohydrate	30 100%	31 100%	WL, TC, LDL-C, HDL-C, TG
Jenkins, (21)	Canada	6	Parallel	39	55	38.5	Overweight/obese; BMI: =27; hyperlipidemia	43% fat, 26% carbohydrate,31% protein	25% fat, 58% carbohydrate, 16% protein	13 68%	10 50%	LDL-C, HDL-C, TC, TG, BG, WL, SBP, DBP
Klemsdal, (44)	Norway	12	Parallel	202	50	42	Overweight/obese; BMI: 28–40; no DM or CVD	35%-40% fat (20% saturated fatty acids), 30%-35% carbohydrate, 25-30% protein	30% fat, 55%–60% carbohydrate, 15% protein	78 78%	86 84%	TC, TG, HDL-C, LDL-C, WL, SBP, DBP, BG
Lim, (45)	American	15	Parallel	60	48.5	22	Overweight/obese; BMI: 28–40; at least one CVD risk factor	60% fat (20% saturated fatty acids), 4% carbohydrate, 35% protein	10% fat (3% saturated fatty acids), 70% carbohydrate, 20% protein	17 63%	18 64%	TC, TG, HDL-C, LDL-C, WL, SBP, DBP, BG
Morgan, (11)	UK	6	Parallel	115	40.7	27	Overweight/obese; BMI: 27–40; no DM	Dr. Atkins’ New Diet Revolution	Rosemary Conley’s diet and fitness plan	33 58%	41 71%	TG, HDL-C, LDL-C, WL, BG
McAuley, (46)	New Zealand	12	Parallel	62	NR	0	Overweight; insulin resistance	Carbohydrate intake =20 g/d in the first 2 weeks, and increasing up to 50 g/day by 8 weeks	<30% fat (<10% saturated fatty acids), >55% carbohydrate, 15% protein	24 75%	24 75%	TC, TG, HDL-C, LDL-C, WL, SBP, DBP, BG
Saslow, (24)	American	12	Parallel	34	59.7	26.5	Overweight/obese; BMI: =25; T2DM	Carbohydrate intake <20–50 g/d	45%–50% carbohydrate	14 88%	15 83%	TG, HDL-C, LDL-C, WL, SBP, DBP
Shai, (53)	Israel	24	Parallel	214	52	94	Obese; BMI: 31; T2DM	Carbohydrate intake <20 g and later 120 g	30% fat (10 % saturated fatty acids), 55–60% carbohydrate, 10–15% protein	85 78%	84 90%	TG, HDL-C, LDL-C, WL, SBP, DBP, BG
Sacks, (52)	American	24	Factorial	403	51	34.5	Overweight/obese; BMI: 33; no DM or unstable CVD	40% fat, 35% carbohydrate, 25% protein	20% fat, 55% carbohydrate, 25% protein	168 83%	157 78%	TC, TG, HDL-C, LDL-C, WL, SBP, DBP, BG
Stern, (47)	American	12	Parallel	132	53.5	82.6	Obese; BMI =35; 83% DM or metabolic syndrome	Carbohydrate intake <30 g/d	To restrict caloric intake by 500 calories per day with <30% of calories from fat	44 69%	43 63%	TC, TG, HDL-C, LDL-C, WL, SBP, DBP, BG
Samaha, (32)	American	6	Parallel	132	54	82.6	Obese; BMI: =35; metabolic syndrome	Carbohydrate intake < 30 g/d	<30% of total energy intake from fat	43 67%	36 53%	TC, TG, HDL-C, LDL-C, WL, BG
Thomson, (33)	American	6	Parallel	43	56.2	0	Overweight/obese; BMI: 25–35; no DM or CVD.	35% Carbohydrate, 25–30% protein, 35-40% fat	55%–60% Carbohydrate,25% fat, 15%–20% protein	19 90%	21 95%	TC, TG, HDL-C, LDL-C, WL, SBP, DBP, BG
Tay, (12)	Australia	6	Parallel	88	50.6	35.2	Overweight/obese; BMI: 33.7; metabolic syndrome	61% fat (20% saturated fat), 4% carbohydrate, 35% protein	30% fat (< 8 % saturated fat), 46% carbohydrate, 24% protein	45 82%	43 80%	TC, TG, HDL-C, LDL-C, WL, SBP, DBP, BG
Wycherley, (49)	Australia	13	Parallel	49	50.0	34.7	Overweight/obese; BMI: 26–43; at least one metabolic syndrome risk factor	61% fat (20% saturated fat), 4% carbohydrate, 35% protein	30% fat (<8% saturated fat), 46% carbohydrate, 24% protein	26 46%	23 38%	TC, TG, HDL-C, LDL-C, WL, SBP, DBP, BG
Wolever, (48)	Canada	12	Parallel	110	59.6	43.3	Overweight/obese; BMI: 24–40; T2DM	40.1% fat, 39.3% carbohydrate, 20.6% protein	Low-glycemic-index (low-fat) diet: 26.5% fat, 51.9% carbohydrates, 21.6% protein,	53 98%	55 98%	TC, TG, HDL-C, LDL-C, WL, SBP, BG
Yamada, (22)	Japan	6	Parallel	24	63.3	50	Obese; BMI: 25.8; T2DM	Carbohydrate intake <70-130 g/d	<25% fat, 50–60% carbohydrate, <20% protein	12 100%	12 100%	TG, HDL-C, LDL-C, WL, SBP, DBP, BG
Yancy, (34)	American	6	Parallel	120	44.9	23.5	Obese; BMI:30–60; hyperlipidemia	Carbohydrate(<20 g/d) decreased to <5 g/d	<30% fat (<10% saturated fatty acids)	45 76%	34 57%	TC, TG, HDL-C, LDL-C, WL, SBP, DBP

^1^LCD, low carbohydrate diets; LFD, Low fat diets; DM, diabetes; T2DM, type 2 diabetes mellitus; CVD, cardiovascular disease; TG, triglyceride; LDL-C, low-density lipoprotein cholesterol; HDL-C, high-density lipoprotein cholesterol; TC, total cholesterol; SBP, systolic blood pressure; DBP, diastolic blood pressure; BG, blood glucose; WL, weight loss; NR, not report.

Although the carbohydrate-restricted diets were prescribed as intake of carbohydrates less than 40% of the total energy intake (E%), actual carbohydrate intakes ranged from 4 to 41.4 E% in 6–11 months, from 4 to 43.5 E% in 12–23 months, and from 30 to 42.5 E% in 24 months. A total of 14 studies were very LCDs (VLCD) (carbohydrate intake ≤50 g/d) and 19 studies were moderate LCDs (MLCD) (carbohydrate intake >50 g/d). Similarly, although the LFD is prescribed as a fat intake of less than 30% of the total energy intake, actual fat intakes ranged from 2.8 to 33 E% in 6–11 months, from 20 to 30.8 E% in 12–23 months, and from 28.4 to 31 E% in 24 months. The completion rates of dietary interventions varied widely, ranging from 38 to 100%. It should be noted that though the intervention of the exercise program was discharged in our present study, a few studies also provided daily exercise volume.

### Quality assessment

Two authors independently assessed the risk of bias in the included studies using the Cochrane Collaboration’s tool. The results of the quality evaluation of the included 33 RCT studies are shown in Supplementary Table 2, which shows that the study qualities of the selected trials were diverse. According to the possibility of bias, the study was assessed as being low risk, moderate risk, or high risk. A total of six studies were evaluated as high risk of bias, three studies were assessed as low risk of bias, and the other studies had a moderate risk of bias. The quality of evidence for outcomes was evaluated as low or very low, and details for the evaluation of the GRADE framework are presented in Supplementary Table 3.

### Effects of low-carbohydrate diets versus low-fat diets on blood lipids

**FIGURE 2:**
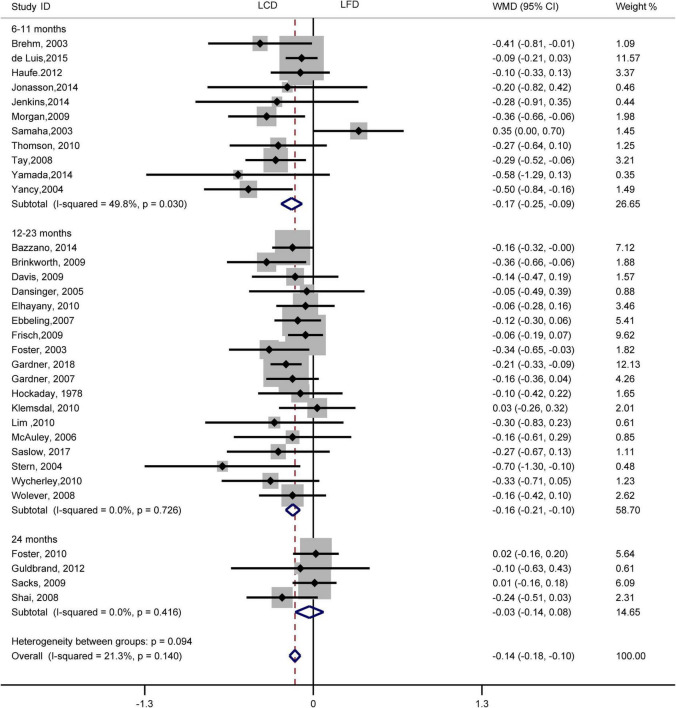
Forest plots showing weight mean differences (WMD) and 95% CI for triglyceride of the low-carbohydrate diets (LCD) in comparison with low-fat diets (LFD).

**FIGURE 3:**
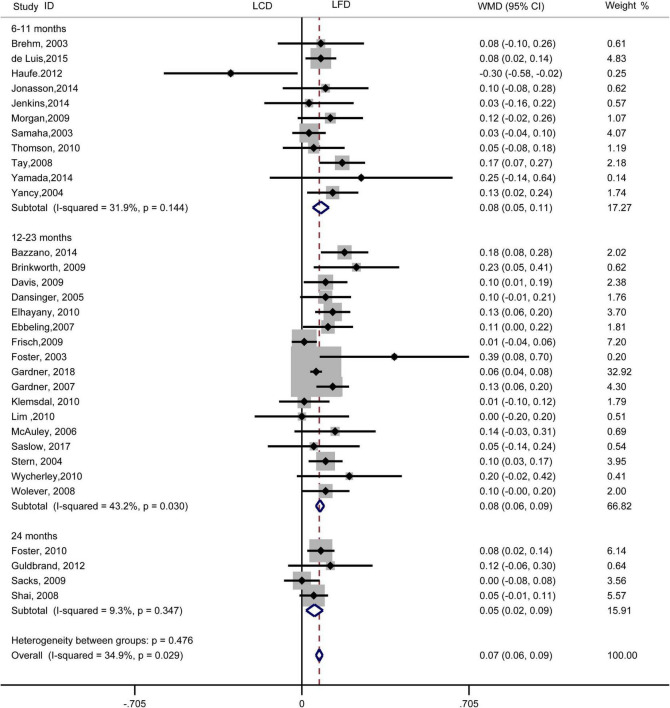
Forest plots showing weight mean differences (WMD) and 95% CI for HDL-cholesterol of the low-carbohydrate diets (LCD) in comparison with low-fat diets (LFD).

**FIGURE 4:**
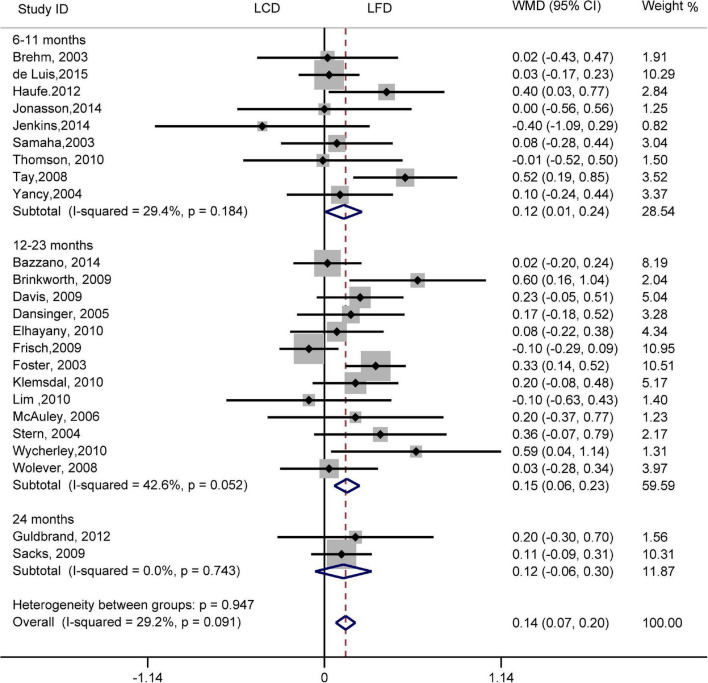
Forest plots showing weight mean differences (WMD) and 95% CI for total cholesterol of the low-carbohydrate diets (LCD) in comparison with low-fat diets (LFD).

**FIGURE 5:**
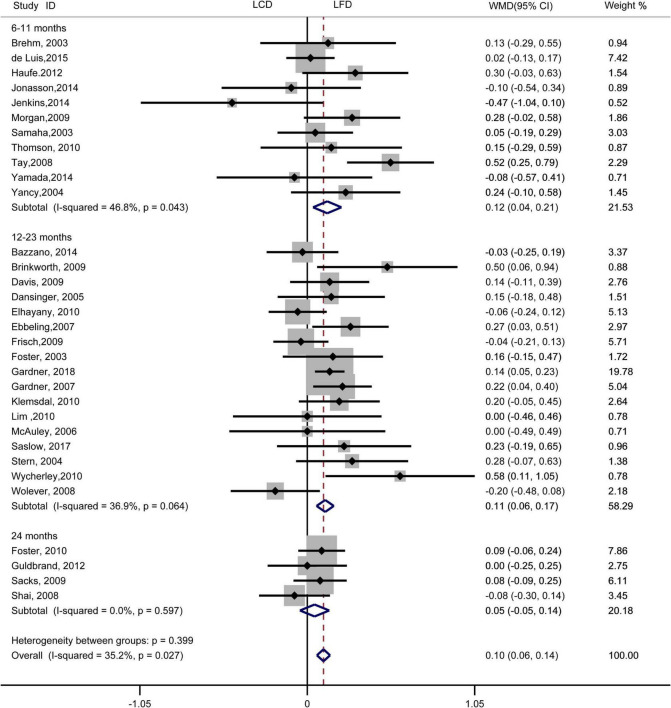
Forest plots showing weight mean differences (WMD) and 95% CI for LDL-cholesterol of the low-carbohydrate diets (LCD) in comparison with low-fat diets (LFD).

The individuals assigned to LCD showed a significantly greater decrease in TG (WMD, –0.14 mmol/L; 95% CI, –0.18 to –0.10 mmol/L; Figure 2) and a significantly greater increase in HDL-C (WMD, 0.07 mmol/L; 95% CI, 0.06–0.09 mmol/L) than the individuals assigned to LFD (Figure 3). However, the pooled effect comparing LCD versus LFD in TC (WMD, 0.14 mmol/L; 95% CI, 0.07 to 0.20 mmol/L; Figure 4) and LDL-C (WMD, 0.10 mmol/L; 95% CI, 0.06 to 0.14 mmol/L) indicates a significantly greater reduction in LFD (Figure 5). It is noteworthy that LCD significantly decreased TG (6–11 months: WMD, –0.17 mmol/L; 95% CI, –0.25 to –0.09 mmol/L; 12–23 months: –0.16 mmol/L; 95% CI, –0.21 to –0.10 mmol/L) and significantly increased HDL-C (6–11 months: WMD, 0.08 mmol/L; 95% CI, 0.05 to 0.11 mmol/L; 12–23 months: 0.08 mmol/L; 95% CI, 0.02 to 0.09 mmol/L) when compared to LFD in 6–23 months, but the reduction effect of LDL-L (6–11 months: WMD, 0.12 mmol/L; 95% CI, 0.04 to 0.21 mmol/L; 12–23 months: 0.11 mmol/L; 95% CI, 0.06 to 0.17 mmol/L) and TC (6–11 months: WMD, 0.12 mmol/L; 95% CI, 0.01 to 0.24 mmol/L; 12–23 months: 0.15 mmol/L; 95% CI, 0.06 to 0.23 mmol/L) was in favor of LFD in 6–23 months. However, these outcomes were not significant differences between the two diets at 24 months. The heterogeneity test showed that four outcomes were of low heterogeneity (TG: *I*^2^= 21,3%, *P* = 0.14; LDL-C: *I*^2^= 35%, *P* = 0.03; HDL-C: *I*^2^= 35%, *P* = 0.03; TC: *I*^2^= 29%, *P* = 0.09).

### Effects of low-carbohydrate diets versus low-fat diets on blood pressure

**FIGURE 6:**
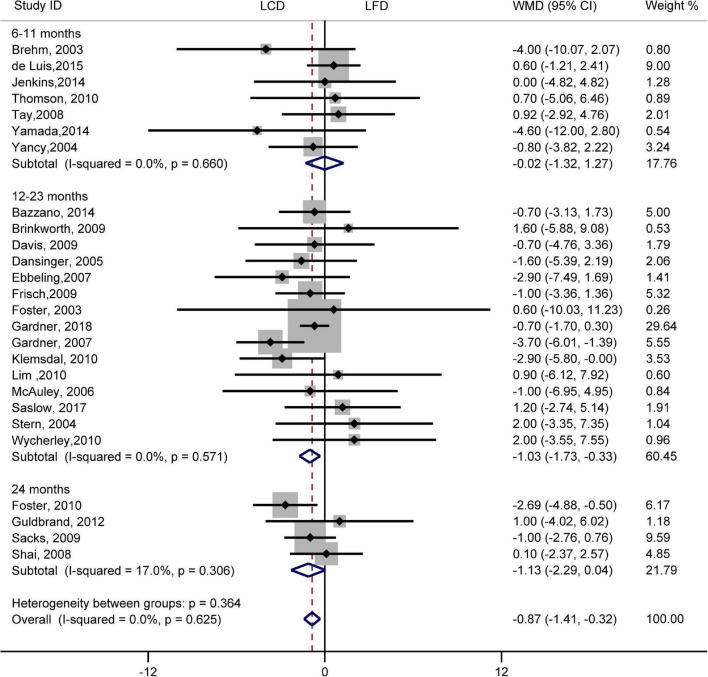
Forest plots showing weight mean differences (WMD) and 95% CI for diastolic blood pressure of the low-carbohydrate diets (LCD) in comparison with low-fat diets (LFD).

There was no difference in the effect of two diets on SBP (WMD, –0.73 mmHg; 95% CI, –1.55 to 0.09 mmHg; *I*^2^ = 21%, *P* = 0.16; Supplementary Figure 1). However, compared with LFD, the decreased DBP was significantly greater in LCD (WMD, –0.87 mmHg; 95% CI, –1.41 to –0.32 mmHg; *I*^2^ = 0%, *P* = 0.62; Figure 6). The difference in the decrease of –1.03 mmHg (95% CI, –1.73 to –0.33 mmHg) also exists in 12–23 months. However, the trend was not significant at 6–11 months or 24 months. The heterogeneity test showed that the results of both SBP and DBP were low heterogeneity.

### Effects of low-carbohydrate diets versus low-fat diets on blood glucose

There was no difference in blood glucose between LCD and LFD (WMD, –0.01 mmol/L; 95% CI, –0.05 to 0.03 mmol/L; Supplementary Figure 2). The heterogeneity test showed that blood glucose was low heterogeneity (*I*^2^= 40%, *P* = 0.02).

### Effects of low-carbohydrate diets versus low-fat diets on weight loss

**FIGURE 7:**
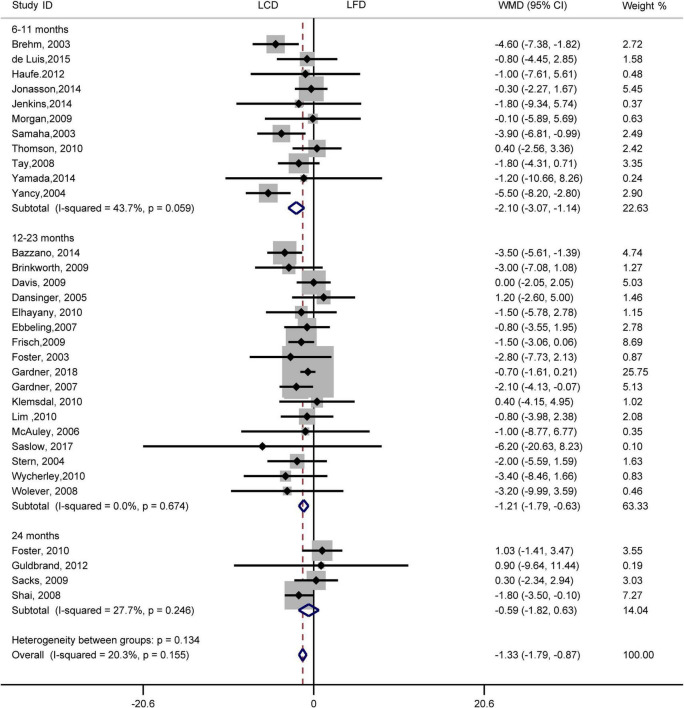
Forest plots showing weight mean differences (WMD) and 95% CI for weight loss of the low-carbohydrate diets (LCD) in comparison with low-fat diets (LFD).

Results indicated that the individuals assigned to LCD showed a greater reduction in weight loss than the individuals assigned to LFD (WMD, –1.33 kg; 95% CI, –1.79 to –0.87 kg; Figure 7). Compared with LFD, the levels of weight loss in LCD decreased by –2.10 kg (95% CI, –3.07 to –1.14 kg) in 6–11 months and –1.21 kg (95% CI, –1.79 to –0.63 kg) in 12–23 months. However, there was no difference in weight loss between the two diets at 24 months. The heterogeneity test showed that weight loss was low heterogeneity (*I*^2^= 20%, *P* = 0.16).

### Subgroup and sensitivity analyses

Subgroup analyses for metabolic risk factors were carried out according to study variables and participant characteristics, including hypertensive status, hyperlipidemia status, diabetic status, energy intake, and proportions of carbohydrates (Supplementary Tables 4–11). Overall, we found that the TC and LDL-C decreased more significantly in LFD. Still, SBP and DBP decreased more obviously in LCD in non-diabetic, non-hypertension, and non-hyperlipidemia participants. However, there were no significant differences between the two diets in participants with diabetes, hypertension, or hyperlipidemia. In identical caloric content and different caloric content subgroups, LCD had a stronger effect on SBP, DBP, and weight loss than LFD in identical caloric content subgroups, but no effect in different caloric content subgroups. When subgroup analyses were conducted based on proportions of carbohydrates, the reduction effect of DBP was favored in LCD in the moderate low carbohydrate subgroup, but not in the subgroup of very low carbohydrate. In the sensitivity analysis, the effects in the results remained unchanged after excluding one study at a time (Supplementary Figures 3–10). Meta-regression was used to explore heterogeneity, and we found that proportions of carbohydrates may be the source of heterogeneity (Supplementary Table 12).

### Publication bias

Results of funnel plots showed that TC, TG, LDL-C, HDL-C, SBP, DBP, blood glucose, and weight loss were symmetric (Supplementary Figures 11–18). Results of the Egger’s tests also showed no significant publication bias (TC: *P* = 0.41; TG: *P* = 0.09; LDL-C: *P* = 0.57; HDL-C: *P* = 0.08; SBP: *P* = 0.61; DBP: *P* = 0.69; blood glucose: *P* = 0.66; weight loss: *P* = 0.38).

## Discussion

Dietary intake, as a rule, follows a pattern of consumption and is one of the main factors that contribute directly to the impaired metabolic risk factors and obesogenic phenotype (54, 55). Numerous studies were performed to compare the effects on metabolic risk factors and weight loss in overweight and obese adults between LCD and LFD (10–15, 20–24, 32–53). However, the studies showed inconsistent results. In our present study, we performed a meta-analysis to the overall existing evidence from randomized controlled trials to compare LCD with LFD. Our results showed different effects on metabolic risk factors and weight loss in adults with overweight or obese between LCD and LFD. Compared with LFD, subjects on LCD had a greater reduction in TG, DBP, weight loss, and greater increases in HDL-C. However, participants on LFD had more decreases in LDL-C and TC. These indicated that we might choose different diets to manage the overweight and obese subjects according to their abnormal metabolic indicators and the need for weight loss.

Our results showed that LCD was more beneficial for improving TG and HDL-C, which was consistent with prior meta-analyses comparing the effect of the two diets in overweight and obese persons (56, 57). Even in healthy subjects in a meta-analysis including eleven randomized controlled trials with 1,369 participants, Nadia et al. also found that HDL-C and triglyceride levels had more favorable changes in LCD (18). The carbohydrate intake and macronutrient composition in LCD were related to the improvement of TG. The production of very low-density lipoprotein triglycerides in the liver is reduced in response to decreased carbohydrate substrate delivery (47). Moreover, the increase of HDL-C on the LCD may cause a greater decrease in TG via downregulation of hepatic scavenger receptor B1 levels because the receptor can bind HDL-C and promote the transportation of cholesterol to the liver (58). We also found that TC and LDL-C decreased significantly in LFD compared with LCD, which is different from previous reports (18, 19). The reasons may be that (1) we included more studies; (2) we included the overweight and obese subjects with or without basic diseases, but the other studies only included healthy persons. In this study, LCD did not cause a significant increase in TC and LDL-C, which may be related to our inclusion of more studies on MLCD. Our results were consistent with Hu et al. (19). However, Mansoor et al. and Lu et al. reported that LCD has adverse effects, which leads to an increase in TC and LDL-C (18, 59). Mansoor et al. only analyzed the effects of VLCD. Noteworthy, the VLCD caused higher levels of TC and LDL-C in many cases (60). The heterogeneity among the included studies was unavoidable. The source of heterogeneity was explored by meta-regression and subgroup analyses. The results found that the proportions of carbohydrates in LCD seem to be part of the source of heterogeneity. The research indicates that very low carbohydrate was related to better blood glucose control and greater weight loss (24), which means that the different content of carbohydrates in interventions may lead to heterogeneity. Moreover, subgroup analyses showed that LCD and LFD had different effects on blood pressure and blood lipids. Among participants with hypertension, hyperlipidemia, and diabetes, the reduction effect of blood pressure, TC, and LDL-C had no significant difference between the two diets. This would imply that the beneficial effects of LCD and LFD on blood pressure, TC, and LDL-C may be at least the same in participants with hypertension, hyperlipidemia, and diabetes. Subgroup analyses on energy intake indicated LCD had a significantly greater reduction of SBP, DBP, and weight loss in identical caloric content subgroups. Still, there was no significant difference in different caloric content subgroups. This difference between the subgroups may be due to the unequal dietary energy between the two diets in the studies because the energy intake of LCD was higher than that of LFD in three studies, and the energy intake of LFD was higher in three separate studies. It should be noted that LCD often increases the proportion of fat, which may cause a higher risk of some cardiovascular diseases or cancers (61). These causal relationships are long-term effects of high-fat diets, but studies included in our present and previous studies often persist from 6 to 24 months. Furthermore, it suggests that moderate replacement of carbohydrates with dietary fats may be a potential method to improve metabolic risk factors and simultaneously prevent increased risk for other diseases. On the other hand, some studies reduced dietary fats and replaced them with carbohydrates such as fruits and grains. However, this replacement did not affect atherogenic dyslipidemia among individuals with metabolic syndrome (62, 63).

Reducing dietary carbohydrates may produce clinical improvements in the management of blood pressure. We found that compared with LFD, individuals assigned to LCD showed a significantly greater reduction in diastolic blood pressure, not in systolic blood pressure, which is similar to a previous study (57). Studies have also shown that LCD with high monounsaturated fatty acids is beneficial for regulating blood pressure in some diseases such as type 2 diabetes (64). However, there was no difference in the improvement of blood pressure between the two diets in two earlier meta-analyses, including overweight and obese subjects (19, 56). The reason for the difference in our results may, at least in part, be more studies were included in the current research.

Both LCD and LFD are beneficial for weight loss. Previous studies have shown that compared with participants on LFD, those on LCD experienced a greater weight loss reduction (56, 65). Moreover, Mansoor et al. found that even in healthy subjects, LCD is more effective for weight loss than LFD (19). Our results are consistent with the effects reported in the above studies, suggesting that the individuals assigned to LCD showed a significantly stronger reduction in weight loss than the individuals assigned to LFD. High fat in LCD can stimulate more secretion of peptide YY, a peptide mainly produced by endocrine L cells, which can reduce appetite and increase satiety (66). Most of the LCDs increase protein intake, thereby increasing subjects’ satiety and reducing eating, which may be related to greater weight loss (33). A further study observed no difference in weight loss between the two diets lasting 24 months. It is similar to the results in an earlier study by Nordmann et al., which found that this different effect on weight loss between the two diets was no longer obvious after 12 months (56). However, the results were inconsistent. Some studies reported that the two diets are at least as effective in weight loss (19).

Reduction of carbohydrate intake has attracted more and more attention in recent years for its potential in health promotion and treatment of diseases, including decreasing body mass, improving fat and carbohydrate metabolism, producing clinical improvements in the management of type 2 diabetes mellitus, and reducing the predicted risk of atherosclerotic cardiovascular disease events (65, 67, 68). However, there are still controversial effects of LCD or the comparison between LCD and LFD (63, 65, 69). Some reasons that may be involved are as follows: (1) the criteria for included subjects are different. The criteria may include only overweight or obese persons or both, while in some studies, the criteria were the different BMI values. In addition, the included participants may be overweight and obese with or without basic diseases or healthy. (2) LCD often increases the percentage of energy from fat. Different fatty acids may have diverse effects. For instance, Abbasnezhad et al. found that LCD with high monounsaturated fatty acids benefits the regulation of blood pressure in some diseases such as type 2 diabetes (64). However, saturated fatty acids have been reported to increase both totals- and LDL-C (70). A study conducted by Sackner-Bernstein et al. showed that LCD is more significant in weight loss and in predicting ASCVD risk in overweight/obese subjects with health or dyslipidemia, but the outcomes were not stratified by follow-up time or different populations (68). Although Chawla et al. performed a stratified analysis of follow-up time, most of the studies were short-term trials (71). They found that LCD is more significant in improving weight loss, HDL-C, and TG within 12 months, but there is a lack of evidence to support the long-term effect of the two diets. This meta-analysis included more studies (over 12 months) and populations, and performed subgroup analyses of different populations and intervention durations to explore the short- and long-term effects of the two diets on metabolic risk factors.

It should be noted that LCD may have some adverse effects. First, LCD often increases the proportion of fat, which may cause a higher risk of some cardiovascular diseases or cancers (61). These causal relationships are long-term effects of high-fat diets, but studies included in our present and previous studies often persist from 6 to 24 months. Second, some meta-analyses based on observational studies have shown that long-term reduction of carbohydrate is related to a significantly increased risk of all-cause mortality (72, 73). Further, the study indicated that the source of food, especially the sources of protein and fat, notably modifies the association between carbohydrate intake and mortality (64). Third, some observational studies reported that in short-term interventional studies in humans, LCD has effects on mood and cognition, such as impaired cognitive function, attenuated performance on a memory-based task, and decreased cognitive processing speed (74–76). However, other studies have shown opposite effects, including having better sleep status, less involvement with mental disorders, and exerting a beneficial effect on depression (77, 78). A systematic review showed that reduction of carbohydrate intake has no stronger effect on psychosocial outcomes than diets of different macronutrient compositions, both in the short- and long-term (79). Thus, further studies are needed to investigate the effects of LCD on psychosocial outcomes.

Several potential limitations should be considered in our study. First, the definitions of LCDs are different. LCD is defined as a total carbohydrate intake of 20–60 g per day or less, or ≤45% of energy from carbohydrates. However, the definition of LFD is consistent, characterized as total fat intake ≤30% of energy from fat. Second, the duration is different, from 6 months to 2 years, and there is no trial lasting for more than 2 years. LCD may produce small short-term improvements in blood glucose control and weight loss, which are not sustained in the long term (80). Thus, the long-term effects of LCD on cardiovascular risk factors and weight loss require further research in the future. Third, only some studies reported the changed types and sources of carbohydrates or fat. Simple or complex carbohydrates have different effects on metabolic risk factors and weight loss (81, 82). Various fatty acids, including saturated, monounsaturated, or polyunsaturated fatty acids, also have diverse effects (83, 84). Therefore, further studies are needed to focus on the various types and sources of carbohydrates or fat in LCD and LFD in the future. Fourth, although we found significant differences in blood lipids, weight loss, and blood pressure between the two diets, most of the outcomes have weak differences, such as DBP, and lack of significant clinically significant. Large-scale clinical studies are needed to confirm the clinical effects of these metabolic risk makers in the future. Finally, the quality of evidence for outcomes ranges from low to very low in this study, not only because of study limitations and indirectness but also because of inconsistency. The dietary trials in participants who are not blinded may be one of the reasons for the low certainty evidence. The quality of evidence for study needs to be improved by well-designed randomized trials in the future.

## Conclusion

In summary, our present meta-analysis found that individuals assigned to LCD showed a significantly greater reduction in TG, diastolic blood pressure, and weight loss, as well as a significant increase in HDL-C. However, LFD was associated with a significantly greater decrease in TC and LDL-C. Moderate restriction of carbohydrate intake in LCD did not cause adverse effects on LDL-C and TC. We also found that LCD was as effective as LFD on weight loss, and metabolic risk factors improvement lasted up to 2 years. However, few large-scale and high-quality studies have analyzed the long-term effects of LCD and LFD on metabolic risk factors. Hence, the long-term clinical efficacy and effects of various sources of carbohydrates or fat in the two diets are still worth further clarification.

## Data availability statement

The original contributions presented in this study are included in the article/Supplementary materials, further inquiries can be directed to the corresponding author.

## Author contributions

LL and JY designed the research. LL and JH were responsible for data acquisition, statistical analysis, and the interpretation of the results. LZ and YH were responsible for providing information and advice on the data synthesis and analysis. SH and JY contributed to the concept and design of the study, provided guidance during study selection, data analysis, draft development, and final submission. All authors read and approved the final manuscript.
