# Supplementary material for: Effects of low-carbohydrate diets versus low-fat diets on metabolic risk factors in overweight and obese adults: A meta-analysis of randomized controlled trials
Source: Front Nutr. 2022 Aug 9;9:935234. doi: 10.3389/fnut.2022.935234 (PMC9397119; doi:10.3389/fnut.2022.935234)

Effects of low carbohydrate diets versus low fat diets on metabolic risk factors in overweight and obese adults: a meta-analysis of randomized controlled trials

Lifu Lei[1,2], Juan Huang[1,2]*, Longlong Zhang[2,3], Yuqin Hong[2,4], Suocheng Hui[1,2#], Jian Yang[1,2#]

Author affiliations:

[1]Department of Clinical Nutrition, The Third Affiliated Hospital of Chongqing Medical University, Chongqing, P.R. China, 410020

[2]Research Center for Metabolic and Cardiovascular Diseases, The Third Affiliated Hospital of Chongqing Medical University, Chongqing, China

[3]Department of Endocrinology, The Third Affiliated Hospital of Chongqing Medical University, Chongqing, P.R. China, 410020

[4]Department of Radiology, The Third Affiliated Hospital of Chongqing Medical University, Chongqing, P.R. China, 410020

* These authors contributed equally to this work.

# Corresponding authors:
Dr. Jian Yang
Department of Clinical Nutrition, The Third Affiliated Hospital of Chongqing Medical University
Chongqing City, 401120
P.R. China
Email: jianyang@hospital.cqmu.edu.cn
and
Dr. Suocheng Hui
Department of Clinical Nutrition, The Third Affiliated Hospital of Chongqing Medical University
Chongqing City, 401120
P.R. China
Email: suochenghui@cqmu.edu.cn

SEARCH STRATEGY FOR PUBMED

#1 Diet, Carbohydrate-Restricted[Mesh]

#2 ((((((((((((((Diet, Carbohydrate Restricted[Title/Abstract]) OR (Diet, Low Carbohydrate[Title/Abstract])) OR (Carbohydrate Diet, Low[Title/Abstract])) OR (Carbohydrate Diets, Low[Title/Abstract])) OR (Diets, Low Carbohydrate[Title/Abstract])) OR (Low Carbohydrate Diets[Title/Abstract])) OR (Carbohydrate-Restricted Diet[Title/Abstract])) OR (Carbohydrate Restricted Diet[Title/Abstract])) OR (Carbohydrate-Restricted Diets[Title/Abstract])) OR (Diets, Carbohydrate-Restricted[Title/Abstract])) OR (Low-Carbohydrate Diet[Title/Abstract])) OR (Diet, Low-Carbohydrate[Title/Abstract])) OR (Diets, Low-Carbohydrate[Title/Abstract])) OR (Low Carbohydrate Diet[Title/Abstract])) OR (Low-Carbohydrate Diets[Title/Abstract]))

#3 #1 OR #2

#4 "Diet, Ketogenic"[Mesh]

#5 ((Ketogenic Diet[Title/Abstract]) OR (Diets, Ketogenic[Title/Abstract])) OR (Ketogenic Diets[Title/Abstract])

#6 #4 OR #5

#7 #6 OR #3

#8 Triglycerides[Mesh]

#9 (Triacylglycerol[Title/Abstract]) OR (Triacylglycerols[Title/Abstract])

#10 #8 OR #9

#11 Lipoproteins, HDL[Mesh]

#12 ((((((((HDL Lipoproteins[Title/Abstract]) OR (Heavy Lipoproteins[Title/Abstract])) OR (Lipoproteins, Heavy[Title/Abstract])) OR (High-Density Lipoproteins[Title/Abstract])) OR (High Density Lipoproteins[Title/Abstract])) OR (Lipoproteins, High-Density[Title/Abstract])) OR (alpha-Lipoproteins[Title/Abstract])) OR (alpha Lipoproteins[Title/Abstract])) OR (alpha-1 Lipoprotein[Title/Abstract])

#13 #11 OR #12

#14 Lipoproteins, LDL[Mesh])

#15 (((((((((LDL Lipoproteins[Title/Abstract]) OR (Low-Density Lipoproteins[Title/Abstract])) OR (Lipoproteins, Low-Density[Title/Abstract])) OR (Low Density Lipoproteins[Title/Abstract])) OR (beta-Lipoproteins[Title/Abstract])) OR (beta Lipoproteins[Title/Abstract])) OR (Low-Density Lipoprotein 1[Title/Abstract])) OR (Low Density Lipoprotein 1[Title/Abstract])) OR (Low-Density Lipoprotein 2[Title/Abstract])) OR (Low Density Lipoprotein 2[Title/Abstract])

#16 #14 OR #15

#17 total cholesterol[Mesh]

#18 Blood Pressure[Mesh]

#19 (((((((Pressure, Blood[Title/Abstract]) OR (Diastolic Pressure[Title/Abstract])) OR (Pressure, Diastolic[Title/Abstract])) OR (Pulse Pressure[Title/Abstract])) OR (Pressure, Pulse[Title/Abstract])) OR (Systolic Pressure[Title/Abstract])) OR (Pressure, Systolic[Title/Abstract])) OR (Pressures, Systolic[Title/Abstract])

#20 #18 OR #19

#21 Blood Glucose[Mesh]

#22 ((Blood Sugar[Title/Abstract]) OR (Sugar, Blood[Title/Abstract])) OR (Glucose, Blood[Title/Abstract])

#23 #21 OR #22

#24 #10 OR #13 OR #16 OR #17 OR #20 OR #23

#25 (((randomized controlled trial) OR controlled clinical trial) OR randomly) OR trials

#26 #7 AND #24 AND #25

SUPPLEMENTARY TABLE 1

Baseline and outcome of thirty - three randomized controlled trials included in the meta-analysis[1]

First author, year (Reference No.)	Group	TC (mmol/L)	TG (mmol/L)	LDL-C (mmol/L)	HDL-C (mmol/L)	SBP (mmHg)	DBP (mmHg)	BG (mmol/L)	WL (kg)
Bazzano, 2014 (1)	LCD	Baseline (5.10 ± 1.10), outcome (0.05 ± 0.68)	Baseline (1.30± 0.60), outcome (-0.23 ± 0.49)	Baseline (3.20 ± 0.90), outcome (-0.08 ± 0.71)	Baseline (1.40 ± 0.30), outcome (0.24 ± 0.31)	Baseline (120.30 ± 12.80), outcome (-0.20 ± 10.40)	Baseline (77.50± 9.00), outcome (-0.50 ± 7.70)	Baseline (5.20 ± 0.60), outcome (0.02 ± 0.79)	Baseline (96.30± 12.70), outcome (-5.30 ± 6.60)
	LFD	Baseline (5.30 ± 1.10), outcome (0.03 ± 0.68)	Baseline (1.40 ± 0.90), outcome (-0.07 ± 0.48)	Baseline (3.20 ± 1.00), outcome (-0.05 ± 0.68)	Baseline (1.50 ± 0.30), outcome (0.06 ± 0.31)	Baseline (124.90 ± 13.80), outcome (-1.30 ± 10.00)	Baseline (79.40 ± 8.30), outcome (0.20 ± 7.40)	Baseline (5.20 ± 0.50), outcome (-0.10 ± 0.54)	Baseline (97.90 ± 13.50), outcome (-1.80 ± 6.50)
Brinkworth, 2009 (2)	LCD	Baseline (5.40 ±1.15), outcome (0.70 ± 1.15)	Baseline (1.67 ± 0.75), outcome (-0.58 ± 0.63)	Baseline (3.20 ± 0.57), outcome (0.60 ± 1.15)	Baseline (1.45 ± 0.29), outcome (0.30 ± 0.40)	Baseline (132.70 ± 13.20), outcome (-13.80 ± 14.36)	Baseline (72.30 ± 10.30), outcome (-6.30 ± 19.90)	Baseline (5.70 ± 0.57), outcome (-0.30 ± 0.06)	Baseline (93.90 ± 15.50), outcome (-14.50 ± 9.80)
	LFD	Baseline (5.40 ± 0.60), outcome (0.10 ± 0.60)	Baseline (1.80 ± 0.84), outcome (-0.22 ± 0.66)	Baseline (3.40 ± 0.60), outcome (0.10 ± 0.60)	Baseline (1.36 ± 0.36), outcome (0.07 ± 0.36)	Baseline (135.2 ± 12.6), outcome (-14.6 ± 12.0)	Baseline (77.10 ± 10.80), outcome (-7.90 ± 9.60)	Baseline (5.60 ± 0.60), outcome (-0.30 ± 0.06)	Baseline (94.50 ± 12.70), outcome (-11.50 ± 9.60)
Brehm, 2003 (3)	LCD	Baseline (5.34± 0.79), outcome (1.11 ± 0.35)	Baseline (1.68 ± 0.70), outcome (1.11 ± 0.35)	Baseline (3.23 ± 0.33), outcome (1.11 ± 0.35)	Baseline (1.34 ± 0.33), outcome (1.11 ± 0.35)	Baseline (116.00 ± 15.00), outcome (1.11 ± 0.35)	Baseline (79.00 ± 12.66), outcome (1.11 ± 0.35)	Baseline (5.51 ± 0.66), outcome (1.11 ± 0.35)	Baseline (91.20 ± 39.40), outcome (1.11 ± 0.35)
	LFD	Baseline (4.77 ± 0.71), outcome (1.11 ± 0.35)	Baseline (1.23 ± 0.49), outcome (1.11 ± 0.35)	Baseline (2.94 ± 0.72), outcome (1.11 ± 0.35)	Baseline (1.26 ± 0.27), outcome (1.11 ± 0.35)	Baseline (115.00 ± 11.18), outcome (1.11 ± 0.35)	Baseline (75.00 ± 8.94), outcome (1.11 ± 0.35)	Baseline (5.06 ± 0.54), outcome (1.11 ± 0.35)	Baseline (92.30 ± 26.83), outcome (1.11 ± 0.35)
De Luis, 2015 (4)	LCD	Baseline (5.34 ± 0.93), outcome (-0.14 ± 0.80)	Baseline (1.38 ± 0.77), outcome (-0.16 ± 0.67)	Baseline (3.32 ± 0.80), outcome (-0.11 ± 0.70)	Baseline (1.41 ± 0.32), outcome (0.05 ± 0.30)	Baseline (128.00 ± 16.80), outcome (-5.6 ± 15.20)	Baseline (81.20 ± 10.50), outcome (-0.90 ± 9.10)	Baseline (5.83 ± 1.01), outcome (-0.29 ± 0.90)	Baseline (92.70 ± 17.80), outcome (-8.70 ± 17.10)
	LFD	Baseline (5.38 ± 1.05), outcome (-0.17 ± 1.00)	Baseline (1.42 ± 0.45), outcome (-0.07 ± 0.45)	Baseline (3.25 ± 0.70), outcome (-0.13 ± 0.70)	Baseline (1.51 ± 0.29), outcome (-0.03 ± 0.30)	Baseline (126.30 ± 16.3), outcome (-2.5 ± 14.7)	Baseline (80.10 ± 6.10), outcome (-1.50 ± 7.70)	Baseline (5.66 ± 0.61), outcome (-0.14 ± 0.50)	Baseline (90.50 ± 18.10), outcome (-7.90 ± 16.80)
Davis, 2009 (5)	LCD	Baseline (4.40 ± 0.83), outcome (0.10 ± 0.76)	Baseline (1.40 ± 0.84), outcome (-0.15 ± 0.88)	Baseline (2.50 ± 0.69), outcome (-0.04 ± 0.63)	Baseline (1.30 ± 0.24), outcome (0.16 ± 0.27)	Baseline (125.00 ± 18.00), outcome (2.00 ± 15.60)	Baseline (73.00 ± 9.00), outcome (-2.90 ± 9.40)	Unavailable	Baseline (93.60 ± 18.00), outcome (-3.10 ± 4.80)
	LFD	Baseline (4.30 ± 0.86), outcome (-0.13 ± 0.70)	Baseline (1.40 ± 0.67), outcome (-0.01 ± 0.86)	Baseline (2.40 ± 0.74), outcome (-0.18 ± 0.66)	Baseline (1.20 ± 0.29), outcome (0.06 ± 0.21)	Baseline (130.00 ± 17.00), outcome (-1.80 ± 22.60)	Baseline (77.00 ± 10.00), outcome (-2.20 ± 11.60)	Unavailable	Baseline (101.00 ± 19.00), outcome (-3.10 ± 5.80)
Dansinger, 2005 (6)	LCD	Baseline (5.53 ± 0.80), outcome (-0.11 ± 0.59)	Baseline (1.72 ± 1.08), outcome (0.01 ± 0.95)	Baseline (3.52 ± 0.80), outcome (-0.18 ± 0.60)	Baseline (1.24 ± 0.41), outcome (0.09 ± 0.18)	Baseline (129.00 ± 17.00), outcome (0.20 ± 12.00)	Baseline (77.00 ± 9.00), outcome (-1.40 ± 7.50)	Baseline (7.06 ± 3.44), outcome (0.08 ±1.67)	Baseline (100.00± 14.00), outcome (-2.10 ± 4.80)
	LFD	Baseline (5.53 ± 0.88), outcome (-0.28 ± 0.54)	Baseline (1.96 ± 1.47), outcome (0.06 ± 0.41)	Baseline (3.52 ± 0.96), outcome (-0.33 ± 0.49)	Baseline (1.16 ± 0.05), outcome (-0.01 ± 0.17)	Baseline (133.00 ± 17.00), outcome (0.50 ± 7.70)	Baseline (76.00 ± 9.00), outcome (0.20 ± 4.60)	Baseline (6.72 ± 3.06), outcome (-0.23 ± 1.67)	Baseline (103.00 ± 15.00), outcome (-3.30 ± 7.30)

SUPPLEMENTARY TABLE 1

Continued[1]

First author, year (Reference No.)	Group	TC (mmol/L)	TG (mmol/L)	LDL-C (mmol/L)	HDL-C (mmol/L)	SBP (mmHg)	DBP (mmHg)	BG (mmol/L)	WL (kg)
Elhayany, 2010 (7)	LCD	Baseline (5.40 ± 0.90), outcome (-0.88 ± 0.93)	Baseline (3.20 ± 0.80), outcome (-1.52 ± 0.68)	Baseline (3.10 ± 0.80), outcome (-0.61 ± 0.49)	Baseline (1.10 ± 0.20), outcome (0.13 ± 0.21)	Unavailable	Unavailable	Baseline (10.10 ± 1.80), outcome (-4.40 ± 1.70)	Baseline (86.70 ± 14.30), outcome (-8.90 ± 13.70)
	LFD	Baseline (5.50 ± 0.80), outcome (-0.96 ± 0.77)	Baseline (3.00 ± 0.70), outcome (-1.46 ± 0.59)	Baseline (3.20 ± 0.80), outcome (-0.55 ± 0.54)	Baseline (1.10± 0.20), outcome (0.00 ± 0.21)	Unavailable	Unavailable	Baseline (10.50 ± 2.00), outcome (-3.50 ± 1.60)	Baseline (85.5 ± 10.60), outcome (-7.40 ± 10.30)
Ebbeling, 2007 (8)	LCD	Unavailable	Baseline (1.26± 1.08), outcome (-0.10 ± 0.36)	Baseline (2.64 ± 0.91), outcome (0.00 ± 0.52)	Baseline (1.47 ± 0.52), outcome (-0.10 ± 0.23)	Baseline (105.00 ± 12.00), outcome (-3.20 ± 14.00)	Baseline (63.00 ± 8.00), outcome (0.00 ± 10.00)	Baseline (4.70 ± 0.44), outcome (0.12 ± 0.44)	Baseline (103.50 ± 17.30), outcome (-2.50 ± 6.00)
	LFD	Unavailable	Baseline (1.42 ± 0.91), outcome (0.02 ± 0.42)	Baseline (3.26 ± 0.88), outcome (-0.27 ± 0.52)	Baseline (1.40 ± 0.37), outcome (-0.21 ± 0.23)	Baseline (108.00 ± 11.00), outcome (1.10 ± 14.00)	Baseline (62.00 ± 9.00), outcome (2.90 ± 10.00)	Baseline (4.88 ± 0.56), outcome (0.08 ± 0.44)	Baseline (103.30 ± 15.10), outcome (-1.70 ± 6.00)
Foster, 2010 (9)	LCD	Baseline (4.88 ± 0.78), outcome (NR ±NR)	Baseline (1.28 ± 0.62), outcome (-0.14 ± 0.77)	Baseline (3.11 ± 0.67), outcome (1.11 ± 0.35)	Baseline (1.20 ± 0.35), outcome (0.20 ± 0.28)	Baseline (124.30 ± 14.10), outcome (-2.68 ± 15.17)	Baseline (73.90 ± 9.40), outcome (-3.19 ± 9.24)	Unavailable	Baseline (103.30 ± 15.5), outcome (-6.34 ± 10.82)
	LFD	Baseline (4.98 ± 0.85), outcome (NR ±NR)	Baseline (1.40 ± 0.83), outcome (-0.16 ± 0.80)	Baseline (3.21 ± 0.76), outcome (-0.12 ± 0.73)	Baseline (1.18 ± 0.30), outcome (0.12 ± 0.23)	Baseline (124.60 ± 15.80), outcome (-2.59 ± 15.67)	Baseline (76.00 ± 9.70), outcome (-0.50 ± 10.32)	Unavailable	Baseline (103.50 ± 14.40), outcome (-7.37 ± 10.98)
Frisch, 2009 (10)	LCD	Baseline (5.50 ± 0.93), outcome (0.03 ± 0.75)	Baseline (1.31 ± 0.56), outcome (-0.10 ± 0.47)	Baseline (3.54 ± 0.80), outcome (0.02 ± 0.65)	Baseline (1.49 ± 0.37), outcome (-0.02 ± 0.21)	Baseline (126.00 ± 13.00), outcome (-5.00 ± 14.00)	Baseline (86.00 ± 8.00), outcome (-3.00 ± 9.00)	Baseline (5.68 ± 1.09), outcome (-0.25 ± 0.75)	Baseline (100.30 ± 15.90), outcome (-5.80 ± 6.10)
	LFD	Baseline (5.54 ± 1.10), outcome (0.13 ± 0.61)	Baseline (1.39 ± 0.65), outcome (-0.04 ± 0.50)	Baseline (3.56 ± 0.91), outcome (0.06 ± 0.59)	Baseline (1.46 ± 0.37), outcome (-0.03 ± 0.17)	Baseline (128.00 ± 14.00), outcome (-1.00 ± 15.00)	Baseline (86.00 ± 8.00), outcome (-2.00 ± 8.00)	Baseline (5.62 ± 0.85), outcome (-0.14 ± 0.46)	Baseline (98.80 ± 16.90), outcome (-4.30 ± 5.10)
Foster, 2003 (11)	LCD	Baseline (5.18 ± 0.87), outcome (0.01 ± 0.33)	Baseline (1.48 ± 1.28), outcome (-0.32 ± 0.27)	Baseline (3.35 ± 0.78), outcome (0.01 ± 0.55)	Baseline (1.21 ± 0.29), outcome (0.47 ± 0.58)	Baseline (120.50 ± 11.00), outcome (-1.60 ± 12.20)	Baseline (74.60 ± 8.5), outcome (-6.10 ± 15.50)	Unavailable	Baseline (98.70± 19.50), outcome (-7.30 ± 7.30)
	LFD	Baseline (5.01 ± 0.83), outcome (-0.32 ± 0.27)	Baseline (1.38 ± 0.93), outcome (-0.20 ± 0.60)	Baseline (3.10 ± 0.78), outcome (-0.15 ± 0.42)	Baseline (1.28 ± 0.32), outcome (0.08 ± 0.39)	Baseline (123.30 ±14.10), outcome (2.90 ± 15.80)	Baseline (77.60 ± 10.80), outcome (-6.70 ± 17.20)	Unavailable	Baseline (98.30 ± 16.40), outcome (-4.50 ± 7.90)
Gardner, 2018 (12)	LCD	Unavailable	Baseline (1.45 ± 1.03), outcome (-0.32 ± 0.64)	Baseline (2.94 ± 0.68), outcome (0.09 ± 0.49)	Baseline (1.29 ± 0.24), outcome (0.07 ± 0.15)	Baseline (122.90 ±12.40), outcome (-3.72 ± 8.60)	Baseline (81.20 ± 9.70), outcome (-2.64 ± 5.30)	Baseline (5.49 ± 0.54), outcome (-0.11 ± 0.49)	Baseline (96.30 ± 15.70), outcome (-5.99 ± 4.80)
	LFD	Unavailable	Baseline (1.45 ± 0.80), outcome (-0.11 ± 0.63)	Baseline (2.89 ± 0.79), outcome (-0.05 ± 0.49)	Baseline (1.28 ± 0.23), outcome (0.01 ± 0.11)	Baseline (122.90 ± 12.50), outcome (-3.18 ± 8.60)	Baseline (81.00 ± 7.30), outcome (-1.94 ± 5.30)	Baseline (5.48 ± 0.48), outcome (-0.20 ± 0.49)	Baseline (97.50 ± 14.70), outcome (-5.29 ± 4.80)

SUPPLEMENTARY TABLE 1

Continued[1]

First author, year (Reference No.)	Group	TC (mmol/L)	TG (mmol/L)	LDL-C (mmol/L)	HDL-C (mmol/L)	SBP (mmHg)	DBP (mmHg)	BG (mmol/L)	WL (kg)
Guldbrand, 2012 (13)	LCD	Baseline (4.50 ± 1.00), outcome (-0.10 ± 1.00)	Baseline (1.70± 1.40), outcome (-0.20 ± 1.20)	Baseline (2.70± 0.90), outcome (-0.30 ± 0.54)	Baseline (1.13 ± 0.33), outcome (0.23 ± 0.40)	Baseline (135.00 ± 15.00), outcome (-9.00 ± 15.00)	Baseline (76.00 ± 11.00), outcome (-5.00 ± 10.00)	Unavailable	Baseline (91.40 ± 19.00), outcome (-2.00 ± 21.00)
	LFD	Baseline (4.30 ± 1.00), outcome (-0.30 ± 1.00)	Baseline (1.80 ± 0.80), outcome (-0.10 ± 0.90)	Baseline (2.40 ± 0.70), outcome (-0.30 ± 0.44)	Baseline (1.09 ± 0.29), outcome (0.11 ± 0.30)	Baseline (136.00 ± 13.00), outcome (-11.00 ± 13.00)	Baseline (77.00 ± 9.00), outcome (-6.00 ± 10.00)	Unavailable	Baseline (98.80 ± 21.00), outcome (-2.90 ± 21.00)
Gardner, 2007 (14)	LCD	Unavailable	Baseline (1.41 ± 0.88), outcome (-0.33 ± 0.67)	Baseline (2.82 ± 0.75), outcome (0.12 ± 0.59)	Baseline (1.37 ± 0.36), outcome (0.13 ± 0.24)	Baseline (118.00 ± 11.00), outcome (-7.60 ± 11.00)	Baseline (75.00 ± 8.00), outcome (-4.40 ± 8.40)	Baseline (5.11 ± 0.50), outcome (-0.10 ± 0.74)	Baseline (86.00 ± 13.00), outcome (-4.70 ± 7.16)
	LFD	Unavailable	Baseline (1.33 ± 0.70), outcome (-0.17 ± 0.52)	Baseline (2.87 ± 0.70), outcome (-0.10 ± 0.49)	Baseline (1.29 ± 0.28), outcome (0.00 ± 0.16)	Baseline (116.00 ± 10.00), outcome (-1.90 ± 7.70)	Baseline (75.00 ± 8.00), outcome (-0.70 ± 6.00)	Baseline (5.17 ± 0.72), outcome (-0.04 ± 0.44)	Baseline (86.00 ± 10.00), outcome (-2.60 ± 5.55)
Haufe, 2012 (15)	LCD	Baseline (4.80 ± 0.70), outcome (0.00 ± 0.80)	Baseline (1.10 ± 0.50), outcome (-0.20 ± 0.50)	Baseline (2.90 ± 0.60), outcome (0.00 ± 0.70)	Baseline (1.60 ± 0.90), outcome (-0.40 ± 0.80)	Unavailable	Unavailable	Baseline (5.27 ± 0.44), outcome (-0.30 ± 0.50)	Baseline (91.00 ± 15.00), outcome (-7.00 ± 15.00)
	LFD	Baseline (4.90 ± 1.00), outcome (-0.40 ± 1.00)	Baseline (1.10 ± 0.60), outcome (-0.10 ± 0.60)	Baseline (3.10 ± 0.90), outcome (-0.30 ± 0.90)	Baseline (1.40 ± 0.60), outcome (-0.10 ± 0.50)	Unavailable	Unavailable	Baseline (5.27 ± 0.44), outcome (-0.30 ± 0.50)	Baseline (91.00 ± 17.00), outcome (-6.00 ± 17.00)
Hockaday, 1978 (16)	LCD	Unavailable	Baseline (1.69 ± 0.88), outcome (-0.10 ± 0.84)	Unavailable	Unavailable	Unavailable	Unavailable	Baseline (10.80 ± 4.26), outcome (-3.40 ± 3.90)	Unavailable
	LFD	Unavailable	Baseline (1.59 ± 0.75), outcome (0.00 ± 0.75)	Unavailable	Unavailable	Unavailable	Unavailable	Baseline (12.50 ± 4.50), outcome (-4.90 ± 4.00)	Unavailable
Jonasson, 2014 (17)	LCD	Baseline (4.50 ± 1.00), outcome (-0.10 ± 1.10)	Baseline (1.70 ± 1.40), outcome (-0.20 ± 1.30)	Baseline (2.70 ± 0.90), outcome (-0.20 ± 0.90)	Baseline (1.10 ± 0.30), outcome (0.10 ± 0.40)	Unavailable	Unavailable	Unavailable	Baseline (NR ± NR), outcome (-4.30 ± 3.60)
	LFD	Baseline (4.30 ± 1.00), outcome (-0.10 ± 1.10)	Baseline (1.80 ± 0.80), outcome (0.00 ± 1.10)	Baseline (2.40 ± 0.70), outcome (-0.10 ± 0.80)	Baseline (1.10 ± 0.30), outcome (0.00 ± 0.30)	Unavailable	Unavailable	Unavailable	Baseline (NR ± NR),, outcome (-4.0 ± 4.10)
Jenkins, 2014 (18)	LCD	Baseline (6.76 ± 1.18), outcome (-0.66 ± 1.10)	Baseline (2.23 ± 1.23), outcome (-0.73 ± 1.00)	Baseline (4.53 ± 0.84), outcome (-0.47± 0.80)	Baseline (1.21 ± 0.32), outcome (0.04 ± 0.30)	Baseline (128.00 ± 9.62), outcome (-5.00 ± 9.60)	Baseline (77.00 ± 6.41), outcome (-1.00± 8.50)	Baseline (5.20 ± 0.43), outcome (-0.60 ± 0.50)	Baseline (83.70 ± 11.21), outcome (-6.80 ± 11.00)
	LFD	Baseline (6.75 ± 0.96), outcome (-0.26 ± 1.10)	Baseline (2.16 ± 1.12), outcome (-0.45 ± 1.00)	Baseline (4.40 ± 0.86), outcome (0.00 ± 1.00)	Baseline (1.36 ± 0.29), outcome (0.01 ± 0.30)	Baseline (122.00 ± 12.45), outcome (-4.00 ± 10.90)	Baseline (75.00 ± 7.26), outcome (-1.00 ± 6.80)	Baseline (5.20 ± 0.52), outcome (-0.60 ± 0.40)	Baseline (85.40 ± 12.76), outcome (-5.00 ± 12.90)

SUPPLEMENTARY TABLE 1

Continued[1]

First author, year (Reference No.)	Group	TC (mmol/L)	TG (mmol/L)	LDL-C (mmol/L)	HDL-C (mmol/L)	SBP (mmHg)	DBP (mmHg)	BG (mmol/L)	WL (kg)
Klemsdal, 2010 (19)	LCD	Baseline (5.80 ± 0.97), outcome (0.00 ± 1.00)	Baseline (1.93 ± 1.21), outcome (-0.26 ± 1.13)	Baseline (3.76 ± 0.94), outcome (0.06 ± 0.85)	Baseline (1.28 ± 0.37), outcome (0.12 ± 0.36)	Baseline (130.00 ± 12.70), outcome (-0.90 ± 14.00)	Baseline (91.00 ± 8.50), outcome (-4.00 ± 10.00)	Baseline (5.62 ± 0.94), outcome (-0.06 ± 0.89)	Baseline (100.00 ± 16.40), outcome (-3.90 ± 17.00)
	LFD	Baseline (6.00 ± 1.04), outcome (-0.20 ± 1.00)	Baseline (1.91 ± 1.13), outcome (-0.29 ± 1.00)	Baseline (3.84 ± 1.01), outcome (-0.14 ± 0.98)	Baseline (1.29 ± 0.37), outcome (0.11 ± 0.41)	Baseline (129.00 ± 15.80), outcome (-2.50 ± 17.00)	Baseline (92.00 ± 9.90), outcome (-1.10 ± 11.00)	Baseline (5.61 ± 0.65), outcome (-0.13 ± 0.72)	Baseline (99.90 ± 14.70), outcome (-4.30 ± 16.00)
Lim, 2010 (20)	LCD	Baseline (5.90 ± 1.00), outcome (-0.40 ± 0.80)	Baseline (1.80 ± 1.00), outcome (-0.20 ± 0.70)	Baseline (3.10± 1.70), outcome (-0.30 ± 0.70)	Baseline (1.30 ± 0.30), outcome (0.10 ± 0.30)	Baseline (129.80 ± 15.1), outcome (-10.60 ± 10.60)	Baseline (77.20 ± 76.40), outcome (-6.60 ± 12.10)	Baseline (5.40 ± 0.60), outcome (0.10 ± 0.30)	Baseline (87.60 ± 2.30), outcome (-2.90 ± 4.90)
	LFD	Baseline (5.70 ± 1.20), outcome (-0.3 ± 0.80)	Baseline (1.60 ± 0.60), outcome (0.10 ± 0.90)	Baseline (2.70 ± 1.90), outcome (-0.30 ± 0.70)	Baseline (1.40 ± 0.40), outcome (0.10 ± 0.30)	Baseline (129.40 ± 12.00), outcome (-6.00 ± 13.30)	Baseline (76.40 ± 9.60), outcome (-7.50 ±8.70)	Baseline (5.30 ± 0.60), outcome (0.30 ± 0.60)	Baseline (89.40 ± 2.50), outcome (-2.10 ±4.70)
Morgan, 2009 (21)	LCD	Unavailable	Baseline (1.65 ± 0.70), outcome (-0.64 ± 0.60)	Baseline (3.72 ± 0.52), outcome (-0.16 ± 0.70)	Baseline (1.22 ± 0.23), outcome (-0.08 ± 0.30)	Baseline (135.00 ± 15.10), outcome (NR ±NR)	Baseline (83.00 ± 10.70), outcome (NR ±NR)	Baseline (5.59 ± 0.45), outcome (-0.29 ± 0.50)	Baseline (92.10 ± 13.60), outcome (-8.90 ± 12.40)
	LFD	Unavailable	Baseline (1.59 ± 0.83), outcome (-0.28 ± 0.70)	Baseline (3.59 ± 0.67), outcome (-0.44 ± 0.60)	Baseline (1.22 ± 0.30), outcome (-0.20 ± 0.30)	Baseline (130.00 ± 14.80), outcome (NR ±NR)	Baseline (82.00 ± 10.30), outcome (NR ±NR)	Baseline (5.66 ± 0.66), outcome (-0.43 ± 0.60)	Baseline (88.20 ± 13.10), outcome (-8.80 ± 12.90)
McAuley, 2006 (22)	LCD	Baseline (5.80 ± 1.10), outcome (-0.20 ± 1.13)	Baseline (1.87 ± 0.82), outcome (-0.47 ± 0.88)	Baseline (3.80 ± 0.90), outcome (-0.10 ± 0.96)	Baseline (1.14 ± 0.29), outcome (0.12 ± 0.34)	Baseline (131.00 ± 14.00), outcome (-5.00 ± 15.00)	Baseline (84.00 ± 10.00), outcome (-4.00 ± 10.00)	Baseline (5.10 ± 0.60), outcome (-0.20 ± 0.67)	Baseline (97.20 ± 10.40), outcome (-5.40 ± 11.00)
	LFD	Baseline (6.00 ± 0.90), outcome (-0.40 ± 0.85)	Baseline (1.88 ± 0.57), outcome (-0.31 ± 0.71)	Baseline (3.90 ± 0.80), outcome (-0.10 ± 0.75)	Baseline (1.16 ± 0.24), outcome (-0.02 ± 0.26)	Baseline (126.00 ± 12.00), outcome (-6.00 ± 12.00)	Baseline (81.00 ± 11.00), outcome (-3.00 ± 11.00)	Baseline (5.00 ± 0.60), outcome (-0.10 ± 0.56)	Baseline (97.60 ± 16.4), outcome (-4.40 ± 16.00)
Saslow, 2017 (23)	LCD	Unavailable	Baseline (1.16 ± 0.41), outcome (-0.11 ± 0.39)	Baseline (2.29 ± 0.56), outcome (0.18 ± 0.58)	Baseline (1.25 ± 0.26), outcome (0.13 ± 0.28)	Baseline (127.10 ± 9.01), outcome (3.20 ± 0.35)	Baseline (77.10 ± 3.46), outcome (-1.50 ± 5.50)	Unavailable	Baseline (99.90 ± 20.00), outcome (-7.90 ± 20.00)
	LFD	Unavailable	Baseline (1.79 ± 0.42), outcome (0.16 ± 0.67)	Baseline (2.54 ± 0.58), outcome (-0.05 ± 0.57)	Baseline (1.18 ± 0.24), outcome (0.08 ± 0.25)	Baseline (129.20 ± 8.21), outcome (1.11 ± 0.35)	Baseline (81.10 ± 5.33), outcome (-2.70 ± 5.30)	Unavailable	Baseline (97.50 ± 19.59), outcome (-1.70 ± 19.60)
Shai, 2009 (24)	LCD	Unavailable	Baseline (2.05 ± 1.32), outcome (-0.27 ± 0.85)	Baseline (3.03 ± 0.89), outcome (-0.08 ± 0.73)	Baseline (0.97 ± 0.22), outcome (0.22 ± 0.18)	Baseline (130.80 ± 15.10), outcome (-3.90 ± 12.80)	Baseline (79.40 ± 9.10), outcome (-0.80 ± 8.70)	Baseline (5.14± 1.58), outcome (0.07 ± 0.55)	Baseline (91.80 ± 14.30), outcome (-4.70 ± 7.00)
	LFD	Unavailable	Baseline (1.77 ± 0.59), outcome (-0.03 ± 1.02)	Baseline (3.04 ± 0.92), outcome (0.00 ± 0.78)	Baseline (1.00 ± 0.25), outcome (0.17 ± 0.23)	Baseline (129.60 ± 13.20), outcome (-4.30 ± 11.80)	Baseline (79.10 ± 9.10), outcome (-0.90 ± 8.10)	Baseline (4.83 ± 1.44), outcome (0.67 ± 0.55)	Baseline (91.30 ± 12.30), outcome (-2.90 ± 4.10)

SUPPLEMENTARY TABLE 1

Continued[1]

First author, year (Reference No.)	Group	TC (mmol/L)	TG (mmol/L)	LDL-C (mmol/L)	HDL-C (mmol/L)	SBP (mmHg)	DBP (mmHg)	BG (mmol/L)	WL (kg)
Sacks, 2009 (25)	LCD	Baseline (5.28 ± 0.91), outcome (-0.05 ± 1.00)	Baseline (1.59 ± 0.96), outcome (-0.26 ± 0.89)	Baseline (3.26 ± 0.80), outcome (-0.05 ± 0.80)	Baseline (1.32 ± 0.41), outcome (0.10 ± 0.41)	Baseline (120.00 ± 15.00), outcome (-0.70 ± 13.00)	Baseline (76.00 ± 10.00), outcome (-1.30 ± 9.00)	Baseline (5.11 ± 0.72), outcome (0.11 ± 0.78)	Baseline (94.00 ± 16.00), outcome (-3.50 ± 13.00)
	LFD	Baseline (5.25 ± 0.93), outcome (-0.16 ± 1.00)	Baseline (1.63 ± 0.89), outcome (-0.27 ± 0.84)	Baseline (3.26 ± 0.83), outcome (-0.13 ± 0.90)	Baseline (1.27 ± 0.34), outcome (0.10 ± 0.36)	Baseline (120.00 ± 13.00), outcome (-1.70 ± 13.00)	Baseline (75.00 ± 9.00), outcome (-0.30 ± 9.00)	Baseline (5.11 ± 0.94), outcome (0.06 ± 0.94)	Baseline (92.00 ± 13.00), outcome (-3.80 ± 14.00)
Stern, 2004 (26)	LCD	Baseline (4.71 ± 1.24), outcome (0.16 ± 1.11)	Baseline (2.27 ± 2.31), outcome (-0.65 ± 1.78)	Baseline (2.90 ± 0.83), outcome (0.18 ± 0.91)	Baseline (1.06 ± 0.26), outcome (-0.03 ± 0.18)	Baseline (133.00 ± 15.00), outcome (1.00 ± 19.00)	Baseline (78.00 ± 11.00), outcome (3.00 ± 15.00)	Baseline (9.21 ± 3.66), outcome (-1.55 ± 2.16)	Baseline (132.00 ± 27.00), outcome (-5.10 ± 8.70)
	LFD	Baseline (5.03 ± 0.75), outcome (-0.20 ± 0.91)	Baseline (1.83 ± 0.88), outcome (0.05 ± 0.96)	Baseline (3.13 ± 0.73), outcome (-0.10 ± 0.75)	Baseline (1.06 ± 0.23), outcome (-0.13 ± 0.16)	Baseline (135.00 ± 16.00), outcome (2.00 ± 15.00)	Baseline (80.00 ± 9.00), outcome (1.00 ± 10.00)	Baseline (8.55 ± 2.78), outcome (-1.17 ± 3.66)	Baseline (130.00 ± 23.00), outcome (-3.10 ± 8.40)
Samaha, 2003 (27)	LCD	Baseline (4.68 ± 1.34), outcome (0.05 ± 0.88)	Baseline (2.12 ± 1.99), outcome (-0.08 ± 0.61)	Baseline (3.05 ± 0.75), outcome (0.13 ± 0.60)	Baseline (1.06 ± 0.28), outcome (0.00 ± 0.13)	Baseline (133.00 ± 15.00), outcome (NR ±NR)	Baseline (78.00 ± 11.00), outcome (NR ±NR)	Baseline (7.11 ± 2.94), outcome (-0.61 ± 1.33)	Baseline (130.00 ± 22.70), outcome (-5.80 ± 8.60)
	LFD	Baseline (4.97 ± 0.78), outcome (-0.03 ± 0.75)	Baseline (1.99 ± 1.35), outcome (-0.43 ± 0.90)	Baseline (2.95 ± 0.93), outcome (0.08 ± 0.47)	Baseline (1.06 ± 0.26), outcome (-0.03 ± 0.18)	Baseline (135.00 ± 16.00), outcome (NR ±NR)	Baseline (80.00 ± 9.00), outcome (NR ±NR)	Baseline (6.89 ± 2.61), outcome (-0.11 ± 1.67)	Baseline (131.80 ± 27.30), outcome (-1.90 ± 4.20)
Thomson, 2010 (28)	LCD	Baseline 5.04 ± 0.66), outcome (-0.14 ± 0.84)	Baseline (1.30 ± 0.51), outcome (-0.35 ± 0.41)	Baseline (2.88 ± 0.60), outcome (-0.05 ± 0.73)	Baseline (1.56 ± 0.37), outcome (0.06 ± 0.21)	Baseline (126.50 ± 13.80), outcome (-0.80 ± 14.00)	Baseline (81.20 ± 11.20), outcome (1.60 ± 11.00)	Baseline (5.43 ± 0.81), outcome (-0.06 ± 0.50)	Baseline (84.90 ± 14.00), outcome (-5.90 ± 4.10)
	LFD	Baseline (5.19 ± 1.02), outcome (-0.13 ± 0.81)	Baseline (1.56 ± 0.61), outcome (-0.08 ± 0.76)	Baseline (3.09 ± 0.88), outcome (-0.20 ± 0.69)	Baseline (1.40 ± 0.46), outcome (0.01 ± 0.21)	Baseline (136.20 ± 21.30), outcome (-8.60 ± 16.30)	Baseline (77.60 ±9.70), outcome (0.90 ± 6.90)	Baseline (5.57 ± 0.68), outcome (-0.07 ± 0.50)	Baseline (83.10 ± 10.50), outcome (-6.30 ± 5.40)
Tay, 2008 (29)	LCD	Baseline (5.40 ± 0.90), outcome (-0.02 ± 0.81)	Baseline (1.60 ± 0.70), outcome (-0.64 ± 0.62)	Baseline (3.20 ± 0.70), outcome (0.06 ± 0.58)	Baseline (1.30 ± 0.40), outcome (0.25 ± 0.28)	Baseline (133.50 ± 14.50), outcome (-12.30 ± 14.10)	Baseline (73.60 ± 11.60), outcome (-4.58 ± 9.78)	Baseline (5.70 ± 0.60), outcome (-0.18 ± 0.40)	Baseline (94.40 ± 15.50), outcome (-11.90 ± 6.30)
	LFD	Baseline (5.40 ± 0.80), outcome (-0.54 ± 0.79)	Baseline (1.80 ± 0.80), outcome (-0.35 ± 0.49)	Baseline (3.30 ± 0.70), outcome (-0.46 ± 0.71)	Baseline (1.40 ± 0.30), outcome (0.08 ± 0.17)	Baseline (136.10 ± 12.60), outcome (-10.80 ± 13.20)	Baseline (77.80 ± 10.10), outcome (-5.50 ± 8.60)	Baseline (5.70 ± 0.80), outcome (-0.21 ± 0.40)	Baseline 95.20 ± 12.60), outcome (-10.10 ± 5.70)
Wycherley, 2010 (30)	LCD	Baseline (5.40 ± 0.97), outcome (0.61 ± 1.20)	Baseline (1.70 ± 0.71), outcome (-0.57 ± 0.61)	Baseline (3.22 ± 0.82), outcome (0.60 ± 1.10)	Baseline (1.41 ± 0.25), outcome (0.28 ± 0.41)	Baseline (134.00 ± 15.30), outcome (-14.00 ± 10.20)	Baseline (73.00 ± 15.30), outcome (-6.00 ± 10.20)	Baseline (5.55 ± 0.46), outcome (0.21 ± 0.36)	Baseline (94.20 ± 16.32), outcome (-14.90 ± 10.70)
	LFD	Baseline (5.59 ± 0.77), outcome (0.02 ± 0.72)	Baseline (1.65 ± 0.67), outcome (-0.24 ± 0.72)	Baseline (3.46 ± 0.72), outcome (0.02 ± 0.48)	Baseline (1.36 ± 0.24), outcome (0.08 ± 0.38)	Baseline (137.00 ± 14.39), outcome (-15.00 ± 14.40)	Baseline (78.00 ± 14.39), outcome (-8.00 ± 9.60)	Baseline (5.56 ± 0.62), outcome (0.29 ± 0.43)	Baseline (97.50 ± 12.95), outcome (-11.50 ± 7.20)

SUPPLEMENTARY TABLE 1

Continued[1]

First author, year (Reference No.)	Group	TC (mmol/L)	TG (mmol/L)	LDL-C (mmol/L)	HDL-C (mmol/L)	SBP (mmHg)	DBP (mmHg)	BG (mmol/L)	WL (kg)
Wolever, 2008 (31)	LCD	Baseline (5.01 ± 0.96), outcome (-0.02 ± 0.80)	Baseline (1.79 ± 0.81), outcome (0.14 ± 0.70)	Baseline (3.02 ± 0.73), outcome (-0.30 ± 0.60)	Baseline (1.16 ± 0.37), outcome (0.05 ± 0.30)	Baseline (127.00 ± 22.05), outcome (0.00 ± 19.20)	Baseline (78.00 ± 14.70), outcome (NR ±NR)	Baseline (7.50 ± 1.47), outcome (0.30 ± 0.98)	Baseline (84.70 ± 19.11), outcome (-0.40 ± 18.20)
	LFD	Baseline (5.09 ± 0.97), outcome (-0.05 ± 0.79)	Baseline (1.87 ± 0.75), outcome (0.30 ± 0.60)	Baseline (3.02 ± 0.97), outcome (-0.10 ± 0.79)	Baseline (1.21 ± 0.22), outcome (-0.05 ± 0.20)	Baseline (124.00 ± 29.93), outcome (5.00 ± 26.80)	Baseline (77.00 ± 14.97), outcome (NR ±NR)	Baseline (7.10 ± 0.75), outcome (0.40 ± 0.47)	Baseline (81.10 ± 18.71), outcome (2.80 ± 17.80)
Yamada, 2014 (32)	LCD	Unavailable	Baseline (1.60 ± 0.86), outcome (-0.66 ± 0.75)	Baseline (2.58 ± 0.73), outcome (-0.12 ± 0.66)	Baseline (1.62 ± 0.44), outcome (0.14 ± 0.52)	Baseline (124.40 ± 10.80), outcome (-1.90 ± 11.40)	Baseline (72.60 ± 6.20), outcome (-6.00 ± 8.30)	Baseline (7.67 ± 2.44), outcome (-0.78 ± 2.10)	Baseline (67.00 ± 15.90), outcome (-2.60 ± 15.00)
	LFD	Unavailable	Baseline (1.75 ± 0.98), outcome (-0.08 ± 0.30)	Baseline (2.90 ± 0.53), outcome (-0.04 ± 0.55)	Baseline (1.55 ± 0.49), outcome (-0.11 ± 0.44)	Baseline (124.90 ± 10.70), outcome (-3.60 ± 11.20)	Baseline (74.80 ± 10.60), outcome (-1.40 ± 10.10)	Baseline (8.61 ± 2.56), outcome (0.44 ± 2.30)	Baseline (68.10 ± 7.70), outcome (-1.40 ± 7.40)
Yancy, 2004 (33)	LCD	Baseline (NR ±NR), outcome (-0.20 ± 0.30)	Baseline (NR ±NR), outcome (-0.80 ± 0.90)	Baseline (NR ±NR), outcome (0.04 ± 0.90)	Baseline (NR ±NR), outcome (0.10 ± 0.30)	Baseline (NR ±NR), outcome (-9.60 ± 14.00)	Baseline (NR ±NR), outcome (-6.00 ± 7.90)	Unavailable	Baseline (NR ±NR), outcome (-12.00 ± 7.00)
	LFD	Baseline (NR ±NR), outcome (-0.30 ± 1.00)	Baseline (NR ±NR), outcome (-0.30 ± 1.00)	Baseline (NR ±NR), outcome (-0.20 ± 1.00)	Baseline (NR ±NR), outcome (-0.03 ± 0.30)	Baseline (NR ±NR), outcome (-7.50 ± 16.00)	Baseline (NR ±NR), outcome (-5.20 ±8.90)	Unavailable	Baseline (NR ±NR), outcome (-6.50 ± 8.00)

[1]All values are expressed as means ± standard deviations; LCD, low carbohydrate diets; LFD, low fat diets; TG, triglyceride; LDL-C, low-density lipoprotein cholesterol; HDL-C, high-density lipoprotein cholesterol; TC, Total cholesterol; SBP, systolic blood pressure; DBP, diastolic blood pressure; BG, blood glucose; WL, weight loss; NR, not report.

SUPPLEMENTARY TABLE 2

Assessment of risk of bias of the studies included in the meta-analysis

Study citation	Random sequence generation	Allocation concealment	Blinding of participants and personnel	Blinding of outcome assessment	Incomplete data outcome	Selective reporting	Other bias
Bazzano, 2014 (1)	Low	Low	Low	Low	Low	Low	Low
Brinkworth, 2009 (2)	Unclear	Unclear	Low	Low	Low	Low	Low
Brehm, 2003 (3)	Low	Unclear	Low	Low	Low	Low	Low
De Luis, 2015 (4)	Unclear	Unclear	Low	Low	Low	Unclear	Unclear
Davis, 2009 (5)	Low	Unclear	Low	Low	Low	Low	Low
Dansinger, 2005 (6)	Unclear	Unclear	Low	Low	Low	Low	Low
Elhayany, 2010 (7)	Unclear	Unclear	Low	Low	High	Low	Unclear
Ebbeling, 2007 (8)	Low	Low	Low	Low	Low	Low	Unclear
Foster, 2010 (9)	Low	Unclear	Low	Low	Low	Low	Unclear
Frisch, 2009 (10)	Low	Unclear	Low	Low	Low	Low	Low
Foster, 2003 (11)	Low	Unclear	Low	Low	Low	Low	Low
Gardner, 2018 (12)	Low	Low	Low	Low	Low	Low	Low
Guldbrand, 2012 (13)	Low	Low	Unclear	Unclear	Low	Low	Low
Gardner, 2007 (14)	Low	Low	Low	Low	Low	Low	Low
Haufe, 2012 (15)	Unclear	Unclear	Low	Low	Low	Low	Unclear
Hockaday, 1978 (16)	Unclear	Unclear	Unclear	Low	Low	Low	Unclear
Jonasson, 2014 (17)	Unclear	Unclear	Low	Low	Low	Low	High
Jenkins, 2014 (18)	Unclear	Low	Low	Low	Low	Low	Unclear
Klemsdal, 2010 (19)	Unclear	Unclear	Low	Low	Unclear	Low	Unclear
Lim, 2010 (20)	Unclear	Unclear	Low	Low	Low	Low	Low
Morgan, 2009 (21)	Unclear	Unclear	Low	Low	Unclear	Low	Unclear
McAuley, 2006 (22)	Unclear	Unclear	Low	Low	Low	Unclear	Low
Saslow, 2017 (23)	Low	Low	High	Unclear	High	Unclear	Low
Shai, 2009 (24)	Low	Unclear	Unclear	Low	Unclear	Low	Low
Sacks, 2009 (25)	Unclear	Low	Low	Low	Unclear	Low	Low
Stern, 2004 (26)	Low	Low	Low	Low	Low	Unclear	Low
Samaha, 2003 (27)	Low	Unclear	Low	Low	Low	Unclear	Low
Thomson, 2010 (28)	Unclear	Unclear	Low	Low	Low	Unclear	Low
Tay, 2008 (29)	Unclear	Unclear	Low	Low	Low	Low	High
Wycherley, 2010 (30)	Unclear	Unclear	Low	Low	Low	Unclear	High
Wolever, 2008 (31)	Low	Low	Unclear	Low	Unclear	Unclear	Low
Yamada, 2014 (32)	Low	Unclear	Unclear	Unclear	Low	Low	Low
Yancy, 2004 (33)	Low	Unclear	Low	Low	High	Low	High

1. Bazzano LA, Hu T, Reynolds K, Yao L, Bunol C, Liu Y, et al. Effects of low-carbohydrate and low-fat diets: a randomized trial. Ann Intern Med. 2014;161(5):309-18.
2. Brinkworth GD, Noakes M, Buckley JD, Keogh JB, Clifton PM. Long-term effects of a very-low-carbohydrate weight loss diet compared with an isocaloric low-fat diet after 12 mo. Am J Clin Nutr. 2009;90(1):23-32.
3. Brehm BJ, Seeley RJ, Daniels SR, D'Alessio DA. A randomized trial comparing a very low carbohydrate diet and a calo-rie-restricted low fat diet on body weight and cardiovascular risk factors in healthy women. J Clin Endocrinol Metab. 2003;88(4):1617-23.
4. de Luis DA, Izaola O, Aller R, de la Fuente B, Bachiller R, Romero E. Effects of a high-protein/low carbohydrate versus a standard hypocaloric diet on adipocytokine levels and insulin resistance in obese patients along 9 months. J Diabetes Complications. 2015;29(7):950-4.
5. Davis NJ, Tomuta N, Schechter C, Isasi CR, Segal-Isaacson CJ, Stein D, et al. Comparative study of the effects of a 1-year dietary intervention of a low-carbohydrate diet versus a low-fat diet on weight and glycemic control in type 2 diabetes. Diabetes Care. 2009;32(7):1147-52.
6. Dansinger ML, Gleason JA, Griffith JL, Selker HP, Schaefer EJ. Comparison of the Atkins, Ornish, Weight Watchers, and Zone diets for weight loss and heart disease risk reduction: a randomized trial. JAMA. 2005;293(1):43-53.
7. Elhayany A, Lustman A, Abel R, Attal-Singer J, Vinker S. A low carbohydrate Mediterranean diet improves cardiovascular risk factors and diabetes control among overweight patients with type 2 diabetes mellitus: a 1-year prospective randomized intervention study. Diabetes Obes Metab. 2010;12(3):204-9.
8. Ebbeling CB, Leidig MM, Feldman HA, Lovesky MM, Ludwig DS. Effects of a low-glycemic load vs low-fat diet in obese young adults: a randomized trial. JAMA. 2007;297(19):2092-102.
9. Foster GD, Wyatt HR, Hill JO, Makris AP, Rosenbaum DL, Brill C, et al. Weight and metabolic outcomes after 2 years on a low-carbohydrate versus low-fat diet: a randomized trial. Ann Intern Med. 2010;153(3):147-57.
10. Frisch S, Zittermann A, Berthold HK, Götting C, Kuhn J, Kleesiek K, et al. A randomized controlled trial on the efficacy of carbohydrate-reduced or fat-reduced diets in patients attending a telemedically guided weight loss program. Cardiovasc Diabetol. 2009;8:36.
11. Foster GD, Wyatt HR, Hill JO, McGuckin BG, Brill C, Mohammed BS, et al. A randomized trial of a low-carbohydrate diet for obesity. N Engl J Med. 2003;348(21):2082-90.
12. Gardner CD, Trepanowski JF, Del Gobbo LC, Hauser ME, Rigdon J, Ioannidis JPA, et al. Effect of low-fat vs low-carbohydrate diet on 12-month weight loss in overweight adults and the association with genotype pattern or insulin secretion: the DIETFITS randomized clinical trial. JAMA. 2018;319(7):667-679.
13. Guldbrand H, Dizdar B, Bunjaku B, Lindström T, Bachrach-Lindström M, Fredrikson M, et al. In type 2 diabetes, randomisation to advice to follow a low-carbohydrate diet transiently improves glycaemic control compared with advice to follow a low-fat diet producing a similar weight loss. Diabetologia. 2012;55(8):2118-27.
14. Gardner CD, Kiazand A, Alhassan S, Kim S, Stafford RS, Balise RR, et al. Comparison of the Atkins, Zone, Ornish, and LEARN diets for change in weight and related risk factors among overweight premenopausal women: the A TO Z Weight Loss Study: a randomized trial. JAMA. 2007;297(9):969-77.
15. Haufe S, Utz W, Engeli S, Kast P, Böhnke J, Pofahl M, et al. Left ventricular mass and function with reduced-fat or re-duced-carbohydrate hypocaloric diets in overweight and obese subjects. Hypertension. 2012;59(1):70-5.
16. Hockaday TD, Hockaday JM, Mann JI, Turner RC. Prospective comparison of modified fat-high-carbohydrate with standard low-carbohydrate dietary advice in the treatment of diabetes: one year follow-up study. Br J Nutr. 1978;39(2):357-62.
17. Jonasson L, Guldbrand H, Lundberg AK, Nystrom FH. Advice to follow a low-carbohydrate diet has a favourable impact on low-grade inflammation in type 2 diabetes compared with advice to follow a low-fat diet. Ann Med. 2014;46(3):182-7.
18. Jenkins DJ, Wong JM, Kendall CW, Esfahani A, Ng VW, Leong TC, et al. Effect of a 6-month vegan low-carbohydrate ('Eco-Atkins') diet on cardiovascular risk factors and body weight in hyperlipidaemic adults: a randomised controlled trial. BMJ Open. 2014;4(2):e003505.
19. Klemsdal TO, Holme I, Nerland H, Pedersen TR, Tonstad S. Effects of a low glycemic load diet versus a low-fat diet in subjects with and without the metabolic syndrome. Nutr Metab Cardiovasc Dis. 2010;20(3):195-201.

20. Lim SS, Noakes M, Keogh JB, Clifton PM. Long-term effects of a low carbohydrate, low fat or high unsaturated fat diet compared to a no-intervention control. Nutr Metab Cardiovasc Dis. 2010;20(8):599-607.
21. Morgan LM, Griffin BA, Millward DJ, DeLooy A, Fox KR, Baic S, et al. Comparison of the effects of four commercially available weight-loss programmes on lipid-based cardiovascular risk factors. Public Health Nutr. 2009;12(6):799-807.
22. McAuley KA, Smith KJ, Taylor RW, McLay RT, Williams SM, Mann JI. Long-term effects of popular dietary approaches on weight loss and features of insulin resistance. Int J Obes (Lond). 2006;30(2):342-9.
23. Saslow LR, Daubenmier JJ, Moskowitz JT, Kim S, Murphy EJ, Phinney SD, et al. Twelve-month outcomes of a randomized trial of a moderate-carbohydrate versus very low-carbohydrate diet in overweight adults with type 2 diabetes mellitus or prediabetes. Nutr Diabetes. 2017;7(12):304.
24. Shai I, Schwarzfuchs D, Henkin Y, Shahar DR, Witkow S, Greenberg I, et al. Weight loss with a low-carbohydrate, mediterranean, or low-fat diet. N Engl J Med. 2008;359(3):229-41.
25. Sacks FM, Bray GA, Carey VJ, Smith SR, Ryan DH, Anton SD, et al. Comparison of weight-loss diets with different compositions of fat, protein, and carbohydrates. N Engl J Med. 2009;360(9):859-73.
26. Stern L, Iqbal N, Seshadri P, Chicano KL, Daily DA, McGrory J, et al. The effects of low-carbohydrate versus conventional weight loss diets in severely obese adults: one-year follow-up of a randomized trial. Ann Intern Med. 2004;140(10):778-85.
27. Samaha FF, Iqbal N, Seshadri P, Chicano KL, Daily DA, McGrory J, et al. A low-carbohydrate as compared with a low-fat diet in severe obesity. N Engl J Med. 2003;348(21):2074-81.
28. Thomson CA, Stopeck AT, Bea JW, Cussler E, Nardi E, Frey G, et al. Changes in body weight and metabolic indexes in overweight breast cancer survivors enrolled in a randomized trial of low-fat vs. reduced carbohydrate diets. Nutr Cancer. 2010;62(8):1142-52.
29. Tay J, Brinkworth GD, Noakes M, Keogh J, Clifton PM. Metabolic effects of weight loss on a very-low-carbohydrate diet com-pared with an isocaloric high-carbohydrate diet in abdominally obese subjects. J Am Coll Cardiol. 2008;51(1):59-67.
30. Wycherley TP, Brinkworth GD, Keogh JB, Noakes M, Buckley JD, Clifton PM. Long-term effects of weight loss with a very low carbohydrate and low fat diet on vascular function in overweight and obese patients. J Intern Med. 2010;267(5):452-61.
31. Wolever TM, Gibbs AL, Mehling C, Chiasson JL, Connelly PW, Josse RG, et al. The Canadian Trial of Carbohydrates in Diabetes (CCD), a 1-y controlled trial of low-glycemic-index dietary carbohydrate in type 2 diabetes: no effect on glycated hemoglobin but reduction in C-reactive protein. Am J Clin Nutr. 2008;87(1):114-25.
32. Yamada Y, Uchida J, Izumi H, Tsukamoto Y, Inoue G, Watanabe Y, et al. A non-calorie-restricted low-carbohydrate diet is effective as an alternative therapy for patients with type 2 diabetes. Intern Med. 2014;53(1):13-9.
33. Yancy WS Jr, Olsen MK, Guyton JR, Bakst RP, Westman EC. A low-carbohydrate, ketogenic diet versus a low-fat diet to treat obesity and hyperlipidemia: a randomized, controlled trial. Ann Intern Med. 2004;140(10):769-77

SUPPLEMENTARY TABLE 3

Assessment of quality of evidence for outcomes using the Grading of Recommendations Assessment, Development and Evaluation (GRADE)

Outcomes	Study limitations	Imprecision	Inconsistency	Indirectness	Publication bias	Quality of evidence (GRADE)
Triglyceride	Few studies (3/33) with low risk of bias, and 6 study with high risk of bias	MD -0.14 lower (-0.18 lower to -0.10 lower)	Relative low level heterogeneity according to I^2 (21.3%) and P (0.14) in direct comparisons	14/33 health adults; similar intervention site; most in double-blind studies; 7/33 long-term intervention	No publication bias	Low (Downgrade by two levels due to study limitations and indirectness)
Total cholesterol	Few studies (1/24) with low risk of bias, and 5 study with high risk of bias	MD 0.14 higher (0.07 higher to 0.20 higher)	Relative low level heterogeneity according to I^2 (29.2%) and P (0.09) in direct comparisons	5/24 health adults; similar intervention site; most in single-blind studies; 4/24 long-term intervention	No publication bias	Low (Downgrade by two levels due to study limitations and indirectness)
LDL-cholesterol	Few studies (3/32) with low risk of bias, and 6 study with high risk of bias	MD 0.10 lower (0.06 lower to 0.14 lower)	Relative moderate level heterogeneity according to I^2 (35.2%) and P (0.03) in direct comparisons	13/32 health adults; similar intervention site; most in double-blind studies; 7/32 long-term intervention	No publication bias	Low (Downgrade by two levels due to study limitations and indirectness)
HDL-cholesterol	Few studies (3/32) with low risk of bias, and 6 study with high risk of bias	MD 0.07 higher (0.06 higher to 0.14 higher)	Relative moderate level heterogeneity according to I^2 (34.9%) and P (0.03) in direct comparisons	13/32 health adults; similar intervention site; most in double-blind studies; 7/32 long-term intervention	No publication bias	Low (Downgrade by two levels due to study limitations and indirectness)

Systolic blood pressure	Few studies (3/27) with low risk of bias, and 3 study with high risk of bias	MD -0.73 lower (-1.55 lower to 0.09 lower)	Relative low level heterogeneity according to I^2 (21.1%) and *P* (0.16) in direct comparisons	8/27 health adults; similar intervention site; half single-blind studies; 7/27 long-term intervention	No publication bias	Low (Downgrade by two levels due to study limitations and indirectness)
Diastolic blood pressure	Few studies (3/26) with low risk of bias, and 3 study with high risk of bias	MD -0.87 lower (-1.41 lower to -0.32 lower)	Low level heterogeneity according to I^2 (0.0%) and *P* (0.63) in direct comparisons	8/26 health adults; similar intervention site; half single-blind studies; 7/26 long-term intervention	No publication bias	Low (Downgrade by two levels due to study limitations and indirectness)
Blood glucose	Few studies (3/26) with low risk of bias, and 3 study with high risk of bias	MD -0.01 lower (-0.05 lower to 0.03 higher)	Moderate level heterogeneity according to I^2 (40.4%) and *P* (0.02) in direct comparisons	8/26 health adults; similar intervention site; most in single-blind studies; 5/26 long-term intervention	No publication bias	Very low (Downgrade by two levels due to study limitations, imprecision and indirectness)
Weight loss	Few studies (3/32) with low risk of bias, and 5 study with high risk of bias	MD -1.33 lower (-1.79 lower to -0.87 lower)	Relative low level heterogeneity according to I^2 (20.3%) and *P* (0.16) in direct comparisons	13/32 health adults; similar intervention site; most in double-blind studies; 7/32 long-term intervention	No publication bias	Low (Downgrade by two levels due to study limitations and indirectness)

SUPPLEMENTARY TABLE 4

Subgroup analyses of total cholesterol

Subgroup	No. of studies	Net change (95% CI)	Test of heterogeneity P^1	I^2(%)	P^2	P^3
Total cholesterol						
Hypertensive status						
Non-hypertension	7	0.17 (0.05, 0.28)	0.25	23.4	0.00	0.22
Hypertension	3	0.04 (-0.14, 0.21)	0.91	0.0	0.67	
Hyperlipidemia status						
Non-hyperlipidemia	9	0.15 (0.06, 0.23)	0.34	11.8	0.00	0.48
Hyperlipidemia	3	0.05 (-0.22, 0.32)	0.37	0.0	0.73	
Diabetic status						
Non-diabetes	10	0.13 (0.04, 0.22)	0.28	18.2	0.00	0.94
Diabetes	5	0.12 (-0.04, 0.28)	0.87	0.0	0.13	
Proportions of carbohydrates						
Very low carbohydrate ≤ 50g	6	0.27 (0.15, 0.39)	0.04	58.2	0.00	0.01
Moderate low carbohydrate > 50g	18	0.08 (0.01, 0.16)	0.71	0.0	0.03	
Energy intake						
Identical caloric content	17	0.08 (0.01, 0.16)	0.13	29.0	0.03	0.11
Different caloric content	5	0.23 (0.07,0.39)	0.61	0.0	0.01	

[1] *P* values for heterogeneity test within subgroups.

[2] *P* values for difference between subgroups.

[3] *P* values for subgroup analysis within each subgroup

SUPPLEMENTARY TABLE 5

Subgroup analyses of triglyceride

Subgroup	No. of studies	Net change (95% CI)	Test of heterogeneity			
			P^1	I^2(%)	P^2	P^3
Triglyceride						
Hypertensive status						
Non-hypertension	10	-0.12 (-0.19, -0.04)	0.63	0.0	0.00	0.05
Hypertension	5	-0.26 (-0.38, -0.41)	0.32	14.8	0.00	
Hyperlipidemia status						
Non-hyperlipidemia	15	-0.13 (-0.18, -0.07)	0.39	5.6	0.00	0.07
Hyperlipidemia	3	-0.36 (-0.61, -0.11)	0.48	0.0	0.01	
Diabetic status						
Non-diabetes	14	-0.13 (-0.19, -0.07)	0.18	25.3	0.00	0.87
Diabetes	8	-0.14 (-0.26, -0.02)	0.93	0.0	0.02	
Proportions of carbohydrates						
Very low carbohydrate ≤ 50g	7	-0.20 (-0.28, -0.11)	0.06	50.5	0.00	0.16
Moderate low carbohydrate > 50g	26	-0.13 (-0.17, -0.08)	0.38	6.0	0.00	
Energy intake						
Identical caloric content	24	-0.15 (-0.19, -0.10)	0.13	24.7	0.00	0.71
Different caloric content	6	-0.18 (-0.31, -0.04)	0.60	0.0	0.01	

[1] *P* values for heterogeneity test within subgroups.

[2] *P* values for difference between subgroups.

[3] *P* values for subgroup analysis within each subgroup

SUPPLEMENTARY TABLE 6

Subgroup analyses of LDL-cholesterol

Subgroup	No. of studies	Net change (95% CI)	Test of heterogeneity			
			P^1	I^2(%)	P^2	P^3
LDL-cholesterol						
Hypertensive status						
Non-hypertension	10	0.10 (0.03, 0.17)	0.39	6.1	0.01	0.99
Hypertension	5	0.10 (-0.04, 0.24)	0.42	0.0	0.16	
Hyperlipidemia status						
Non-hyperlipidemia	15	0.10 (0.04, 0.15)	0.54	0.0	0.00	0.66
Hyperlipidemia	3	0.04 (-0.21, 0.29)	0.11	54.7	0.76	
Diabetic status						
Non-diabetes	14	0.11 (0.05, 0.18)	0.41	3.9	0.00	0.04
Diabetes	7	-0.02 (-0.12, 0.09)	0.55	0.0	0.73	
Proportions of carbohydrates						
Very low carbohydrate ≤ 50g	7	0.18 (0.08, 0.28)	0.02	62.2	0.00	0.07
Moderate low carbohydrate > 50g	25	0.08 (0.04, 0.13)	0.23	16.7	0.00	
Energy intake						
Identical caloric content	23	0.10 (0.05, 0.14)	0.01	44.5	0.00	0.89
Different caloric content	6	-0.11 (-0.03, 0.24)	0.19	33.3	0.12	

[1] *P* values for heterogeneity test within subgroups.

[2] *P* values for difference between subgroups.

[3] *P* values for subgroup analysis within each subgroup

SUPPLEMENTARY TABLE 7

Subgroup analyses of HDL-cholesterol

Subgroup	No. of studies	Net change (95% CI)	Test of heterogeneity			
			P[1]	I^2(%)	*P*[2]	*P*[3]
HDL-cholesterol						
Hypertensive status						
Non-hypertension	10	0.10 (0.07, 0.13)	0.77	0.0	0.00	0.32
Hypertension	5	0.13 (0.07, 0.19)	0.59	0.0	0.00	
Hyperlipidemia status						
Non-hyperlipidemia	15	0.08 (0.06, 0.11)	0.05	41.2	0.00	0.48
Hyperlipidemia	3	0.11 (0.03, 0.20)	0.63	0.0	0.01	
Diabetic status						
Non-diabetes	14	0.09 (0.06, 0.12)	0.04	43.2	0.00	0.39
Diabetes	7	0.11 (0.07, 0.16)	0.97	0.0	0.00	
Proportions of carbohydrates						
Very low carbohydrate ≤ 50g	7	0.13 (0.09, 0.17)	0.19	31.3	0.00	0.00
Moderate low carbohydrate > 50g	25	0.06 (0.05, 0.08)	0.23	16.9	0.00	
Energy intake						
Identical caloric content	23	0.07 (0.06, 0.09)	0.05	34.9	0.00	0.46
Different caloric content	6	0.09 (0.04, 0.14)	0.16	37.4	0.00	

[1]*P* values for heterogeneity test within subgroups.

[2] *P* values for difference between subgroups.

[3] *P* values for subgroup analysis within each subgroup

SUPPLEMENTARY TABLE 8

Subgroup analysis of systolic blood pressure

Subgroup	No. of studies	Net change (95% CI)	Test of heterogeneity			
			P^1	I^2(%)	P^2	P^3
Systolic blood pressure						
Hypertensive status						
Non-hypertension	10	-2.33 (-3.86, -0.80)	0.18	28.3	0.00	0.03
Hypertension	4	0.92 (-1.65, 3.50)	0.35	9.2	0.48	
Hyperlipidemia status						
Non-hyperlipidemia	13	-0.81 (-1.97, 0.36)	0.03	48.6	0.18	0.90
Hyperlipidemia	3	-1.05 (-4.70, 2.60)	0.81	0.0	0.57	
Diabetic status						
Non-diabetes	12	-1.22 (-2.43, -0.00)	0.04	46.5	0.05	0.07
Diabetes	5	2.11 (-1.27, 5.48)	0.48	0.0	0.22	
Proportions of carbohydrates						
Very low carbohydrate ≤ 50g	7	-0.12 (-1.95, 1.71)	0.89	0.0	0.90	0.46
Moderate low carbohydrate > 50g	20	-0.89 (-1.81, 0.03)	0.05	36.8	0.06	
Energy intake						
Identical caloric content	19	-1.00 (-1.89, -0.10)	0.12	28.4	0.03	0.18
Different caloric content	5	1.33 (-1.97, 4.62)	0.40	1.2	0.43	

[1] *P* values for heterogeneity test within subgroups.

[2] *P* values for difference between subgroups.

[3] *P* values for subgroup analysis within each subgroup

SUPPLEMENTARY TABLE 9

Subgroup analysis of diastolic blood pressure

Subgroup			Test of heterogeneity			
	No. of studies	Net change (95% CI)	*P*[1]	I^2(%)	*P*[2]	*P*[3]
Diastolic blood pressure						
Hypertensive status						
Non-hypertension	10	-1.41 (-2.43, -0.38)	0.19	27.4	0.01	0.57
Hypertension	4	-0.82 (-2.57, 0.93)	0.73	0.0	0.36	
Hyperlipidemia status						
Non-hyperlipidemia	13	-1.14 (-1.93, -0.34)	0.25	18.8	0.01	0.70
Hyperlipidemia	3	-0.64 (-2.99, 1.71)	0.96	0.0	0.59	
Diabetic status						
Non-diabetes	12	-1.24 (-2.06, 0.42)	0.33	11.6	0.00	0.35
Diabetes	4	-0.05 (-2.39, 2.29)	0.55	0.0	0.97	
Proportions of carbohydrates						
Very low carbohydrate ≤ 50g	7	-0.99 (-2.26, 0.29)	0.55	0.0	0.13	0.84
Moderate low carbohydrate > 50g	19	-0.84 (-1.44, -0.24)	0.51	0.0	0.01	
Energy intake						
Identical caloric content	19	-0.74 (-1.33, -0.15)	0.65	0.0	0.01	0.35
Different caloric content	4	0.41 (-1.90, 2.71)	0.80	0.0	0.73	

[1] *P* values for heterogeneity test within subgroups.

[2] *P* values for difference between subgroups.

[3] *P* values for subgroup analysis within each subgroup

SUPPLEMENTARY TABLE 10

Subgroup analysis of blood glucose

Subgroup	No. of studies	Net change (95% CI)	Test of heterogeneity			
			P[1]	I^2(%)	*P*[2]	*P*[3]
Blood glucose						
Hypertensive status						
Non-hypertension	6	-0.09 (-0.18, 0.01)	0.53	0.0	0.07	0. 04
Hypertension	4	0.09 (-0.05, 0.24)	0.46	0.0	0.20	
Hyperlipidemia status						
Non-hyperlipidemia	11	-0.03 (-0.10, 0.04)	0.06	44.0	0.41	0.93
Hyperlipidemia	2	-0.04 (-0.26, 0.18)	0.66	0.0	0.72	
Diabetic status						
Non-diabetes	11	-0.02 (-0.08, 0.05)	0.44	0.0	0.65	0.10
Diabetes	4	-0.24 (-0.49, 0.02)	0.01	73.7	0.07	
Proportions of carbohydrates						
Very low carbohydrate ≤ 50g	4	0.02 (-0.08, 0.12)	0.65	0.0	0.70	0.50
Moderate low carbohydrate > 50g	22	-0.02 (-0.07, 0.03)	0.01	47.4	0.47	
Energy intake						
Identical caloric content	21	-0.01 (-0.06, 0.04)	0.00	50.8	0.67	0.63
Different caloric content	4	-0.05 (-0.20, 0.10)	0.90	0.0	0.53	

[1]*P* values for heterogeneity test within subgroups.

[2] *P* values for difference between subgroups.

[3] *P* values for subgroup analysis within each subgroup

SUPPLEMENTARY TABLE 11

Subgroup analysis of weight loss

Subgroup	No. of studies	Net change (95% CI)	Test of heterogeneity			
			P[1]	I^2(%)	*P*[2]	*P*[3]
Weight loss						
Hypertensive status						
Non-hypertension	10	-1.17 (-2.14, -0.20)	0.229	23.3	0.02	0. 04
Hypertension	5	-2.92 (-4.31, -1.53)	0.046	56.7	0.00	
Hyperlipidemia status						
Non-hyperlipidemia	15	-1.33 (-2.05, -0.61)	0.16	26.8	0.00	0.01
Hyperlipidemia	3	-4.69 (-7.10, -2.27)	0.41	0.0	0.00	
Diabetic status						
Non-diabetes	14	-1.83 (-2.65, -1.01)	0.03	47.6	0.00	0.08
Diabetes	7	-0.44 (-1.73, 0.85)	0.94	0.0	0.50	
Proportions of carbohydrates						
Very low carbohydrate ≤ 50g	7	-2.51 (-3.60, -1.42)	0.03	57.6	0.00	0.02
Moderate low carbohydrate > 50g	25	-1.07 (-1.58, -0.57)	0.73	0.0	0.00	
Energy intake						
Identical caloric content	23	-1.48 (-1.98, -0.99)	0.08	30.4	0.00	0.41
Different caloric content	6	-0.77 (-2.39, 0.85)	0.84	0.0	0.35	

[1]*P* values for heterogeneity test within subgroups.

[2] *P* values for difference between subgroups.

[3] *P* values for subgroup analysis within each subgroup

SUPPLEMENTARY TABLE 12

Meta regression analyses of mean difference of outcomes in follow-up duration, hypertensive status, hyperlipidemia status, diabetic status, energy intake, proportions of carbohydrates

Outcome	No of studies	Effect size (95% CI)	*P*[1]
	Follow-up duration		
Triglyceride (mmol/L)	33	0.06 (-0.02, 0.14)	0.12
Total cholesterol (mmol/L)	24	0.00 (-0.10, 0.11)	0.95
LDL- cholesterol (mmol/L)	32	-0.05 (-0.14, 0.04)	0.29
HDL- cholesterol (mmol/L)	32	-0.01 (-0.03, 0.01)	0.34
Systolic blood pressure (mmHg)	27	0.93 (-0.79, 2.65)	0.28
Diastolic blood pressure (mmHg)	26	-0.52 (-1.44, 0.39)	0.25
Blood glucose (mmol/L)	26	0.01 (-0.11, 0.13)	0.87
Weight loss (kg)	32	0.86 (-0.14, 1.86)	0.09
	Hypertension status		
Triglyceride (mmol/L)	15	-0.14 (-0.31, 0.02)	0.08
Total cholesterol (mmol/L)	10	-0.12 (-0.40, 0.16)	0.36
LDL- cholesterol (mmol/L)	15	0.00 (-0.18, 0.18)	0.99
HDL- cholesterol (mmol/L)	15	0.03 (-0.38, 0.10)	0.34
Systolic blood pressure (mmHg)	14	2.94 (-1.38, 7.26)	0.16
Diastolic blood pressure (mmHg)	14	0.61 (-2.25, 3.48)	0.65
Blood glucose (mmol/L)	10	0.18 (-0.02, 0.38)	0.08
Weight loss (kg)	15	-1.35 (-4.21, 1.52)	0.33
	Hyperlipidemia status		
Triglyceride (mmol/L)	18	-0.23 (-0.51, 0.04)	0.09
Total cholesterol (mmol/L)	12	-0.11 (-0. 45, 0.24)	0.51
LDL- cholesterol (mmol/L)	18	-0.06 (-0. 35, 0.23)	0.68
HDL- cholesterol (mmol/L)	18	-0.03 (-0. 09, 0.14)	0.62
Systolic blood pressure (mmHg)	16	-0.34 (-5. 41, 4.73)	0.89
Diastolic blood pressure (mmHg)	16	-0.58 (-2.50, 3.65)	0.69
Blood glucose (mmol/L)	13	-0.01 (-0.34, 0.32)	0.95
Weight loss (kg)	18	-3.11 (-6. 49, 0.28)	0.07
	Diabetic status		
Triglyceride (mmol/L)	22	-0.01 (-0.15, 0.14)	0.90
Total cholesterol (mmol/L)	15	-0.01 (-0.21, 0.21)	0.95
LDL- cholesterol (mmol/L)	21	-0.13 (-0.26, 0.00)	0.05
HDL- cholesterol (mmol/L)	21	-0.02 (-0.04, 0.09)	0.50
Systolic blood pressure (mmHg)	17	-3.18 (-1.36, 7.73)	0.16
Diastolic blood pressure (mmHg)	16	-1.22 (-1.76, 4.19)	0. 40
Blood glucose (mmol/L)	15	-0.23 (-0.60, 0.14)	0.20
Weight loss (kg)	21	-1.05 (-1. 40, 3. 50)	0.38
	Proportions of carbohydrates		
Triglyceride (mmol/L)	33	0.08 (-0.04, 0.21)	0.17
Total cholesterol (mmol/L)	24	-0.19 (-0.39, 0.02)	0.03
LDL- cholesterol (mmol/L)	32	-0.14 (-0.28, 0.01)	0.07

HDL- cholesterol (mmol/L)	32	-0.07 (-0.13, 0.02)	0.01
Systolic blood pressure (mmHg)	27	-0.56 (-3.27, 2.15)	0.67
Diastolic blood pressure (mmHg)	26	0.03 (-1.59, 1.65)	0.97
Blood glucose (mmol/L)	26	-0.06 (-0.23, 0.11)	0. 45
Weight loss (kg)	32	-1.39 (-0.14, 2.91)	0.07
	Energy balance		
Triglyceride (mmol/L)	30	0.01 (-0.18, 0.21)	0.76
Total cholesterol (mmol/L)	22	0.13 (-0.08, 0.35)	0.21
LDL- cholesterol (mmol/L)	29	0.01 (-0.18, 0.21)	0.89
HDL- cholesterol (mmol/L)	29	0.00 (-0.06, 0.07)	0.97
Systolic blood pressure (mmHg)	24	2.30 (-1.71, 6.31)	0.25
Diastolic blood pressure (mmHg)	23	1.14 (-1.38, 3.67)	0.36
Blood glucose (mmol/L)	25	-0.02 (-0.25, 0.21)	0.84
Weight loss (kg)	29	0.70 (-1. 48, 2.88)	0.52

[1]*P* values of meta regression analysis based on restricted maximum likelihood model.

SUPPLEMENTARY FIGURE 1. Forest plots for systolic blood pressure

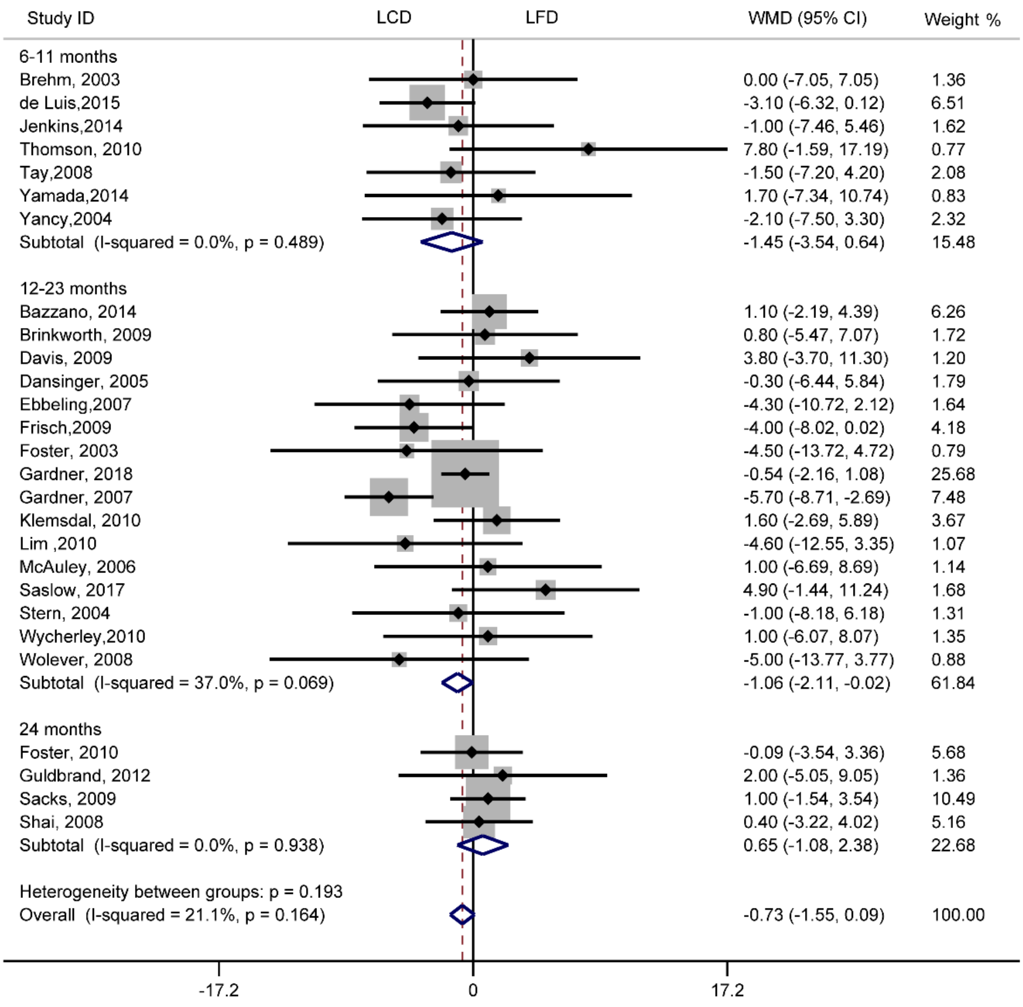

SUPPLEMENTARY FIGURE 2. Forest plots for blood glucose

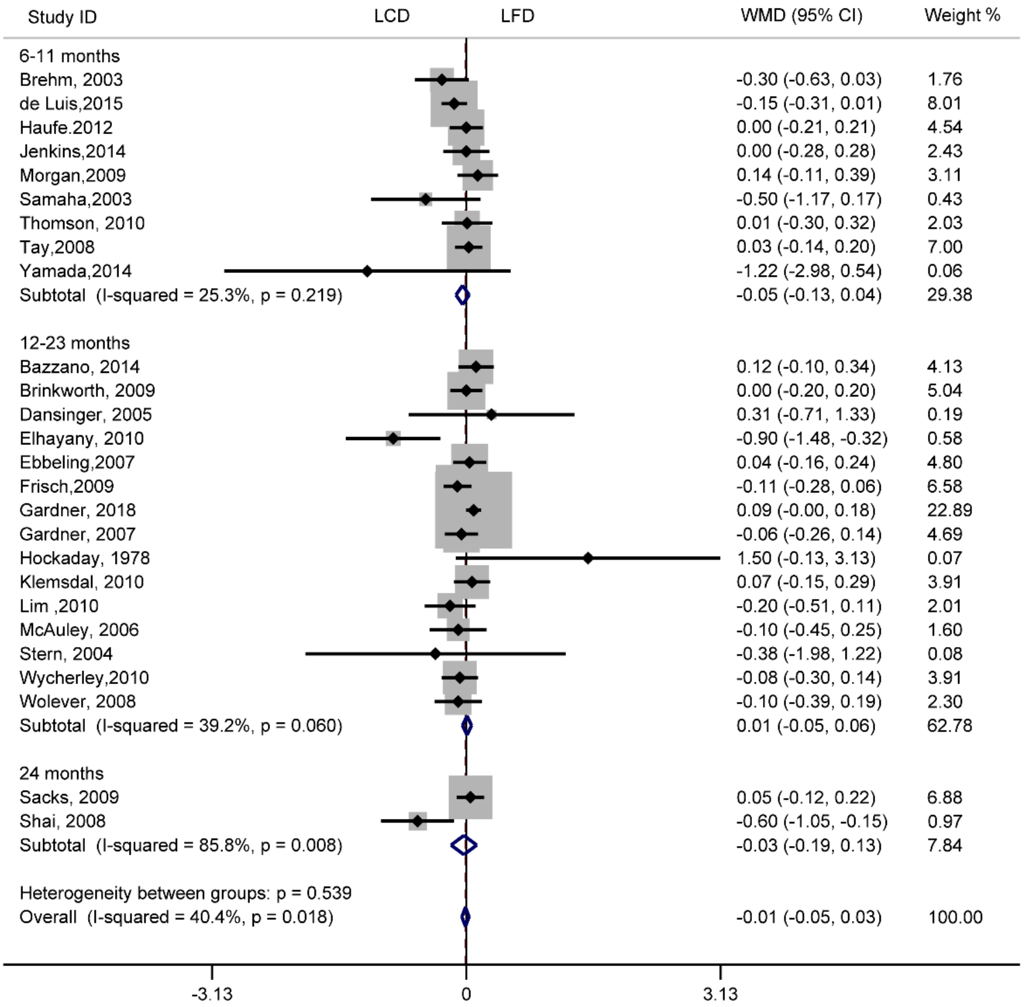

SUPPLEMENTARY FIGURE 3. Sensitivity analyses for triglycerides

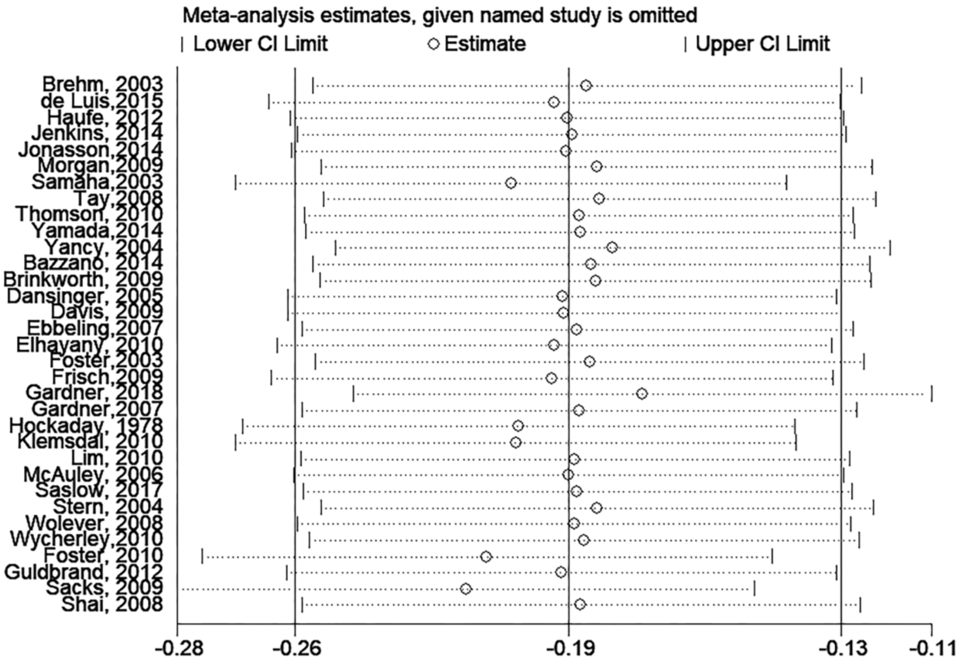

SUPPLEMENTARY FIGURE 4. Sensitivity analyses for total cholesterol

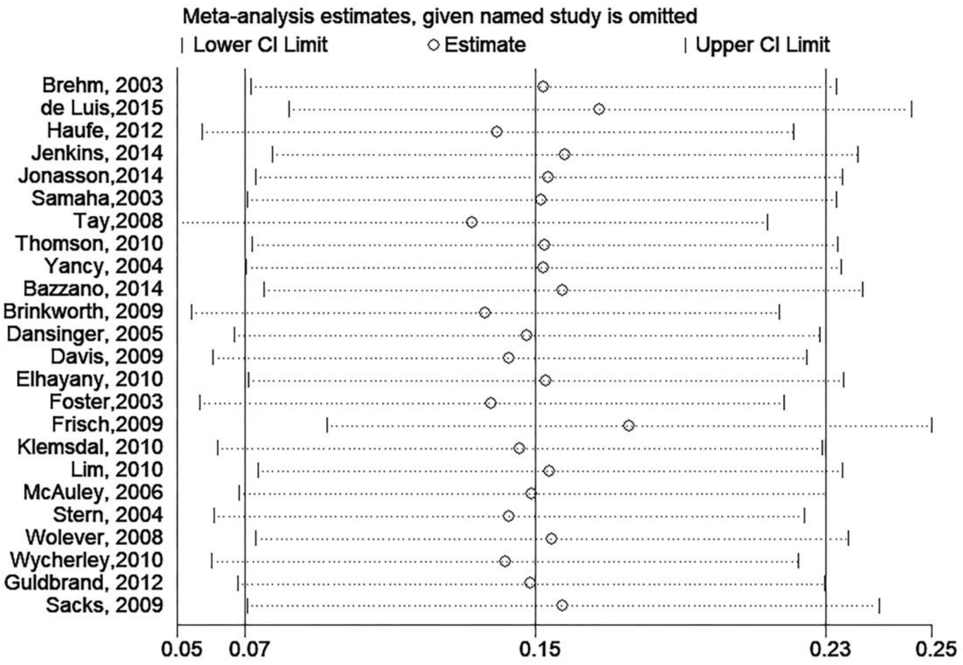

SUPPLEMENTARY FIGURE 5. Sensitivity analyses for LDL-cholesterol

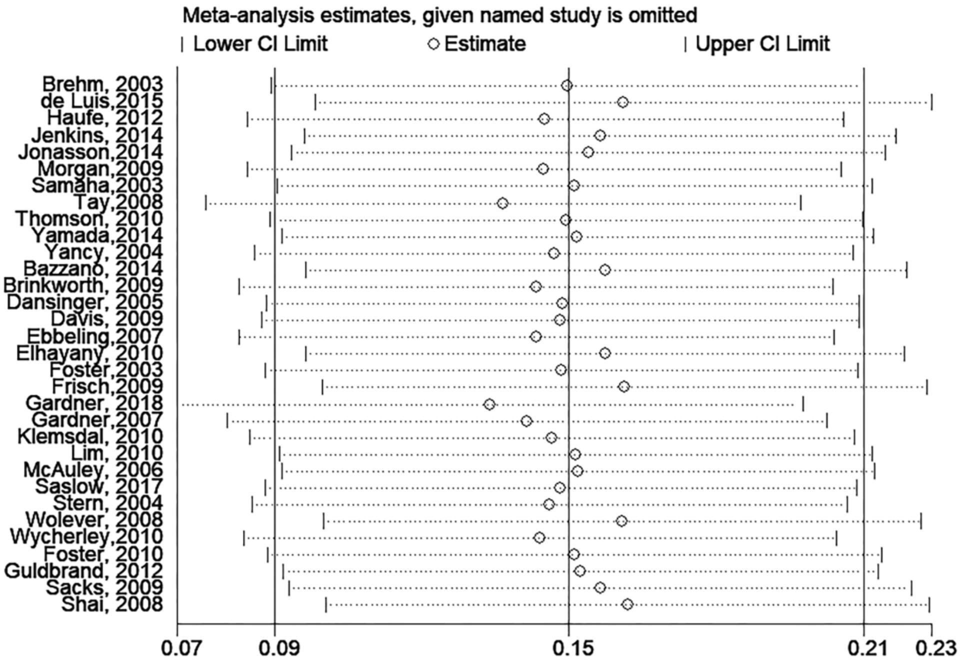

SUPPLEMENTARY FIGURE 6. Sensitivity analyses for HDL-cholesterol

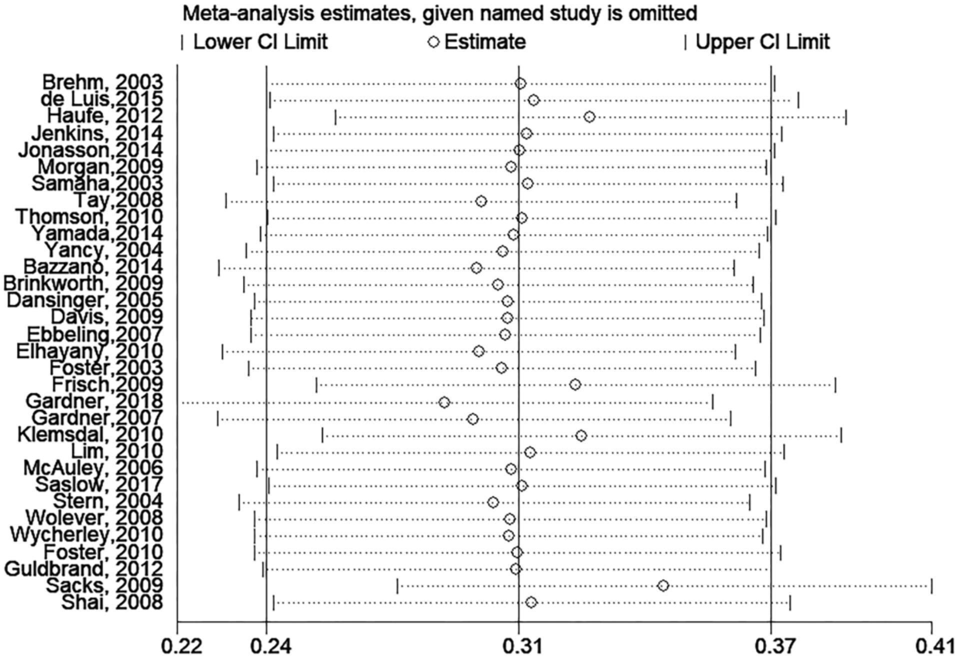

SUPPLEMENTARY FIGURE 7. Sensitivity analyses for systolic blood pressure

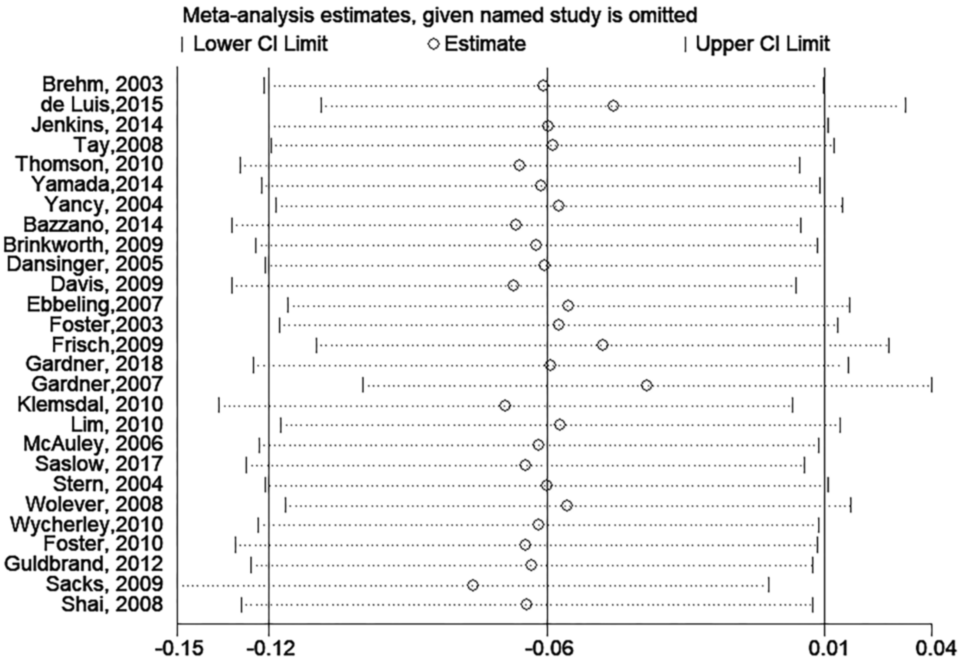

SUPPLEMENTARY FIGURE 8. Sensitivity analyses for diastolic blood pressure

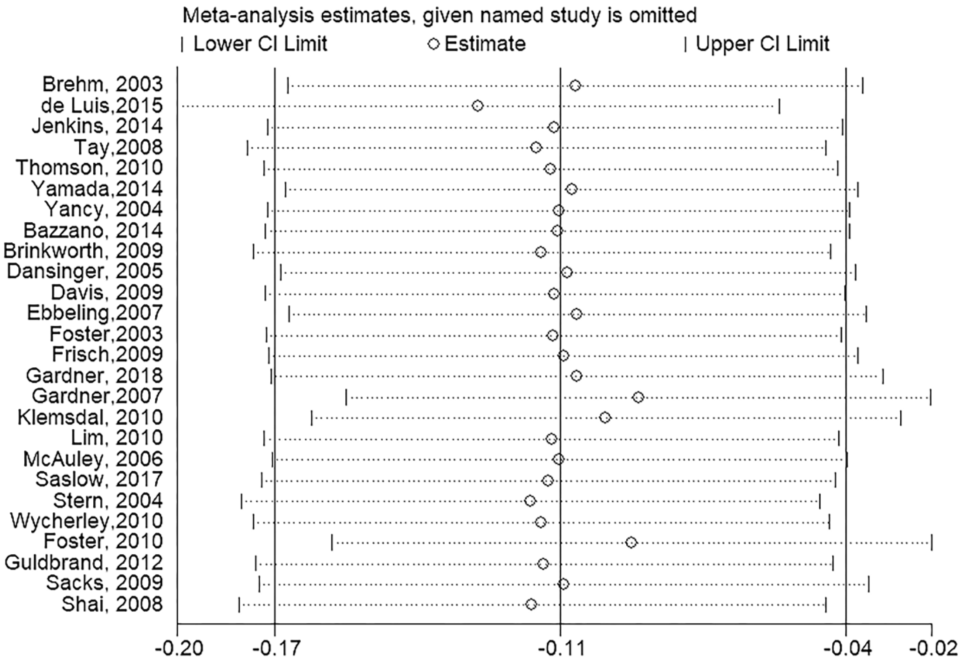

SUPPLEMENTARY FIGURE 9. Sensitivity analyses for blood glucose

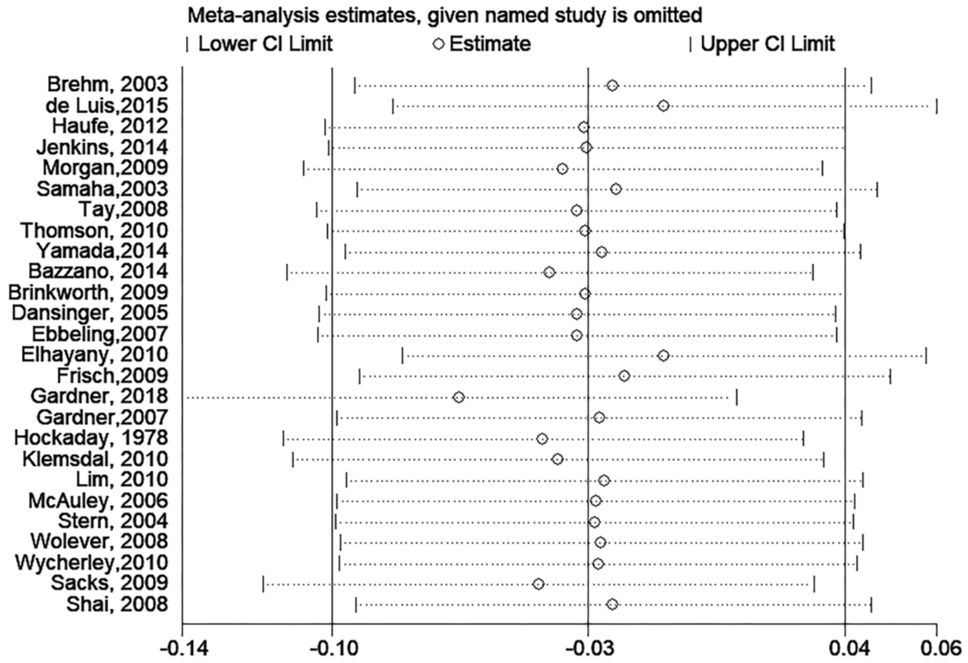

SUPPLEMENTARY FIGURE 10. Sensitivity analyses for weight loss

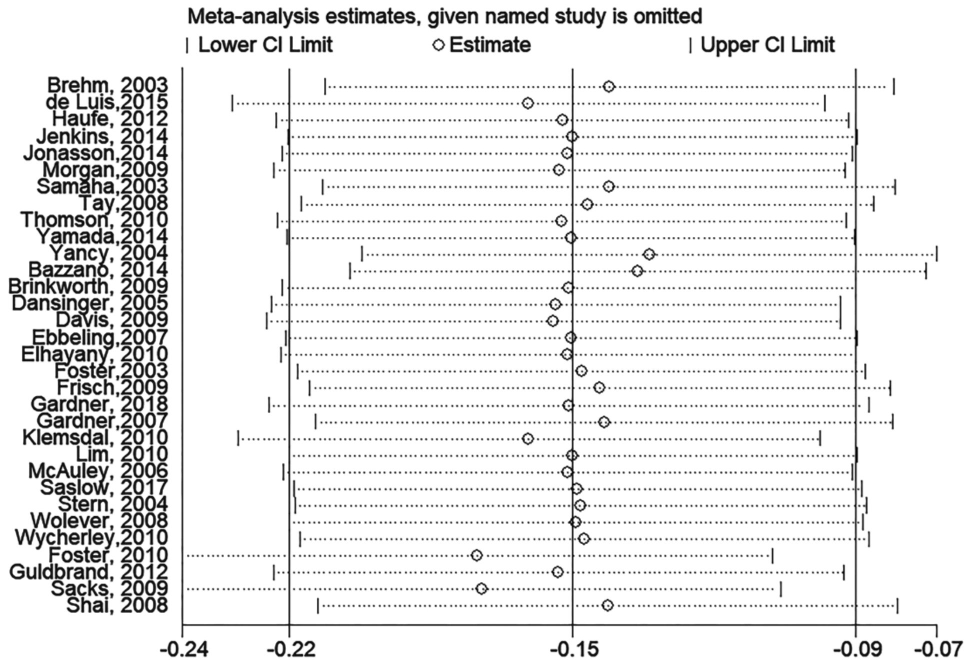

SUPPLEMENTARY FIGURE 11. Funnel plots for triglycerides

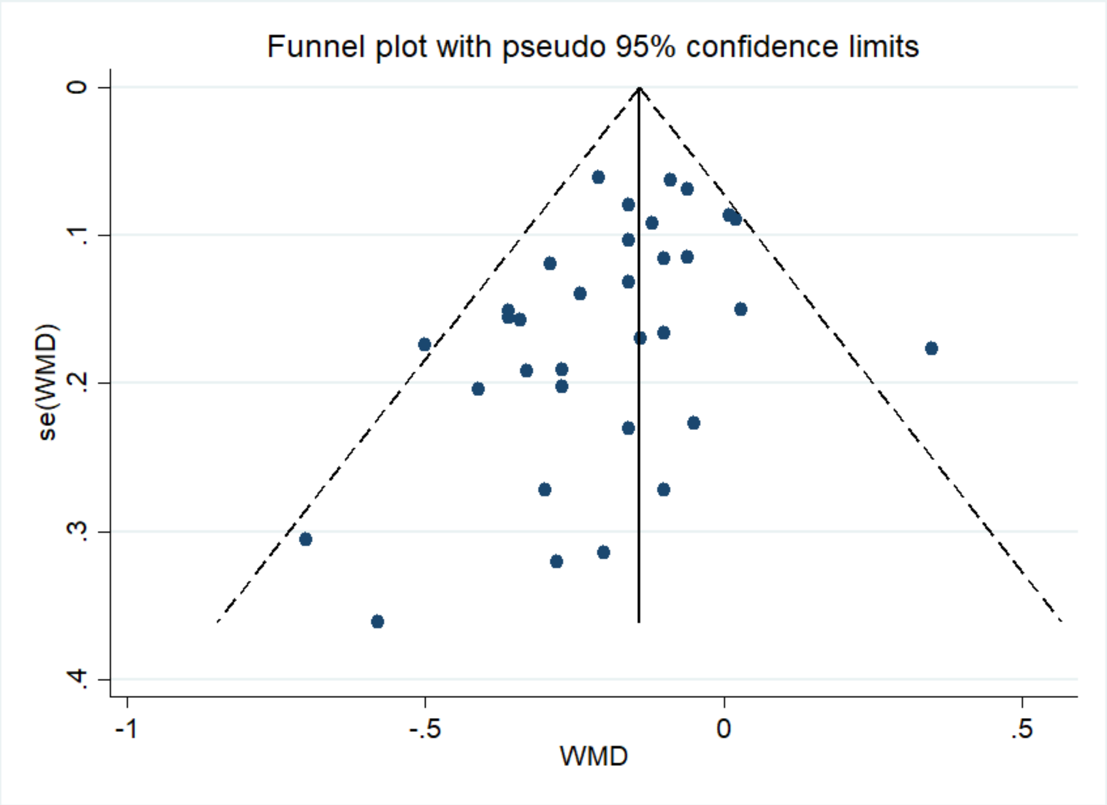

SUPPLEMENTARY FIGURE 12. Funnel plots for total cholesterol

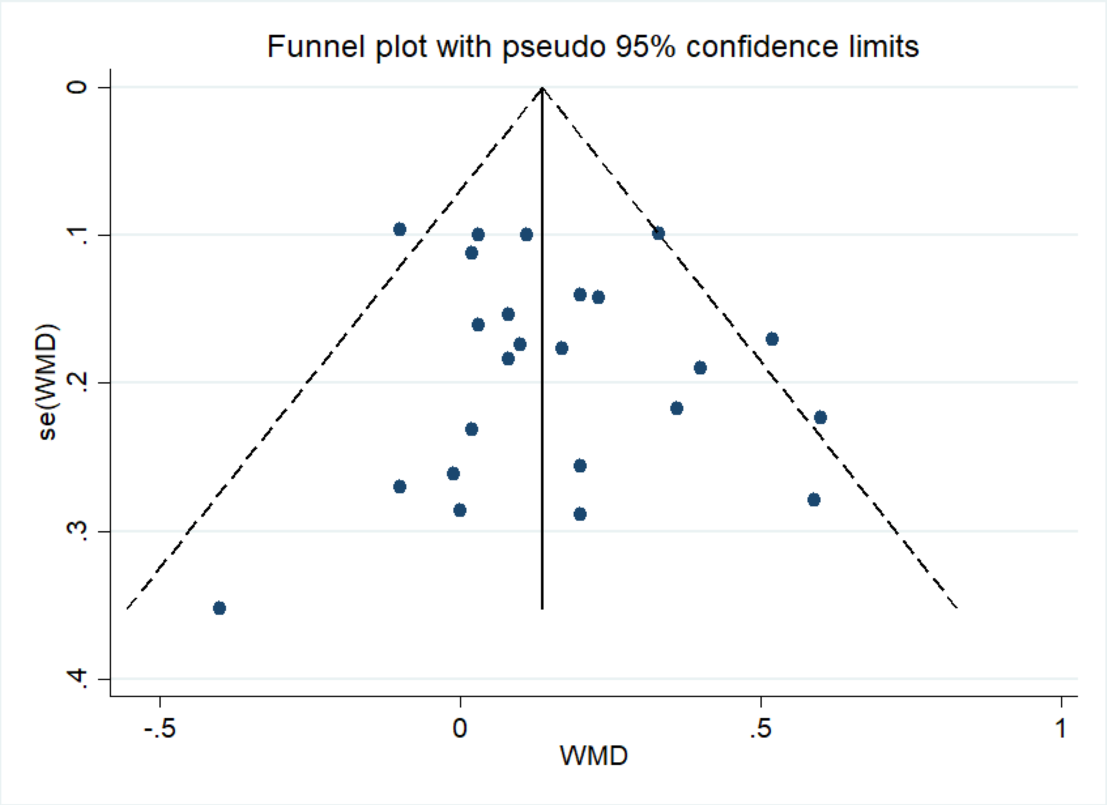

SUPPLEMENTARY FIGURE 13. Funnel plots for LDL-cholesterol

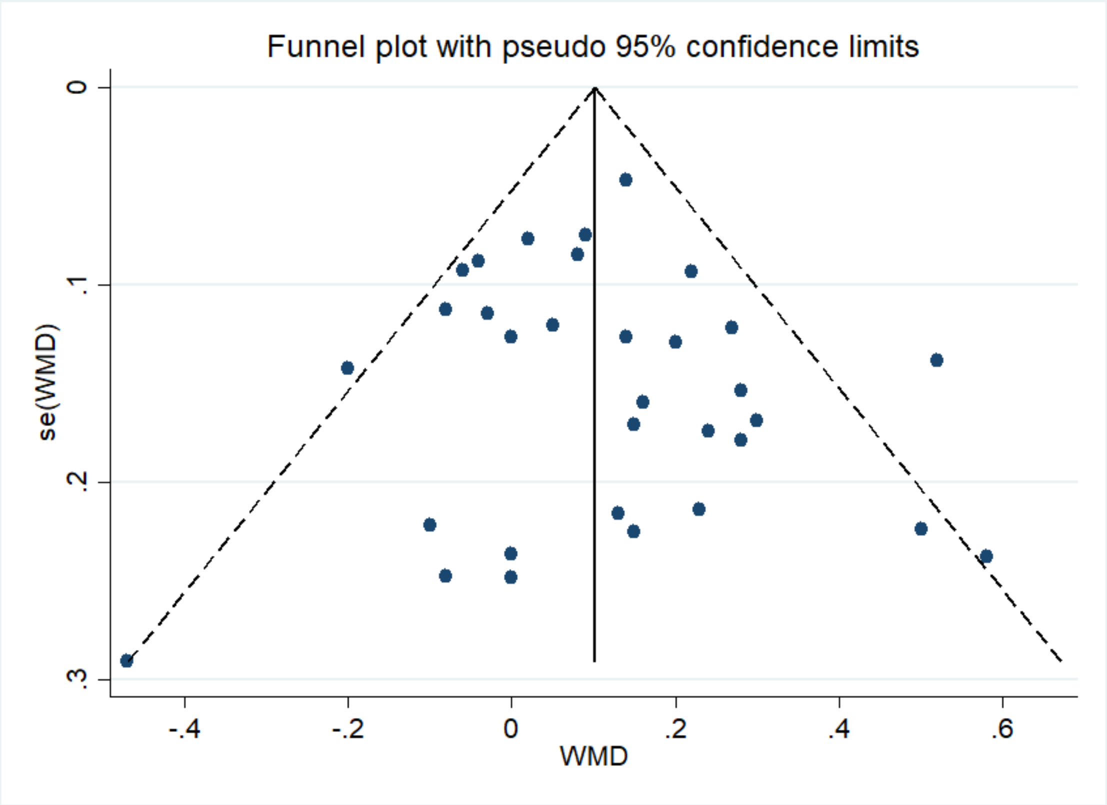

SUPPLEMENTARY FIGURE 14. Funnel plots for HDL-cholesterol

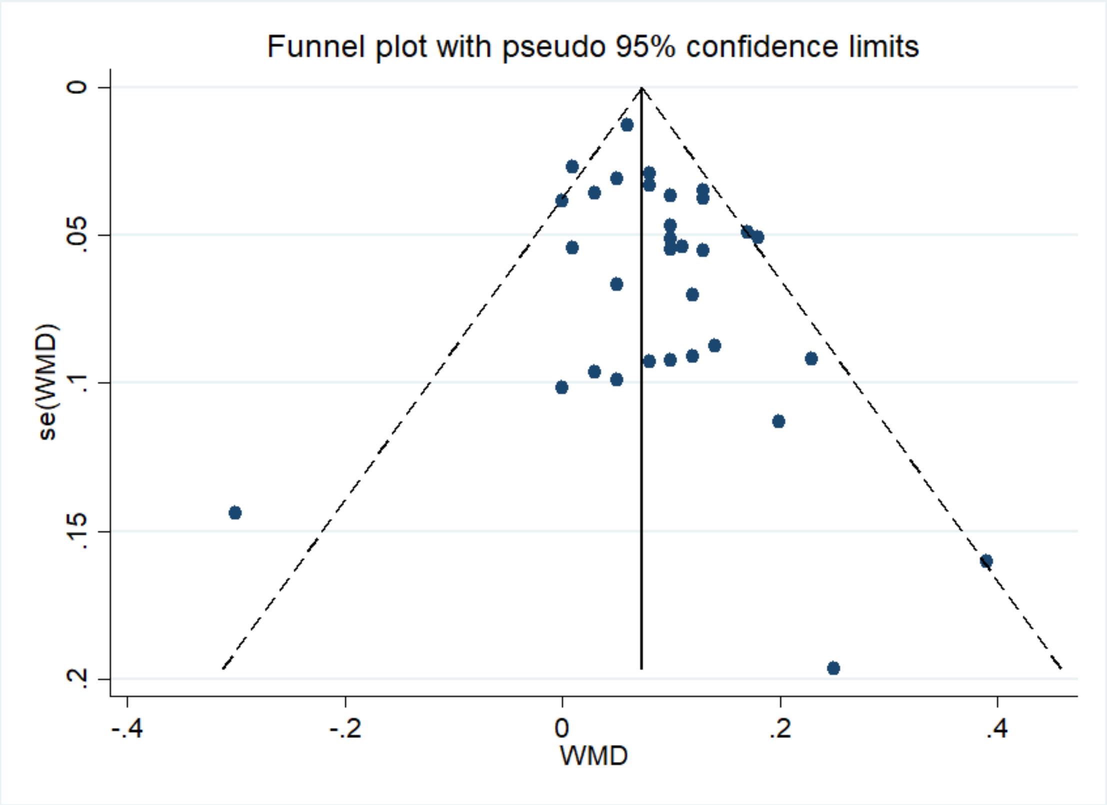

SUPPLEMENTARY FIGURE 15. Funnel plots for systolic blood pressure

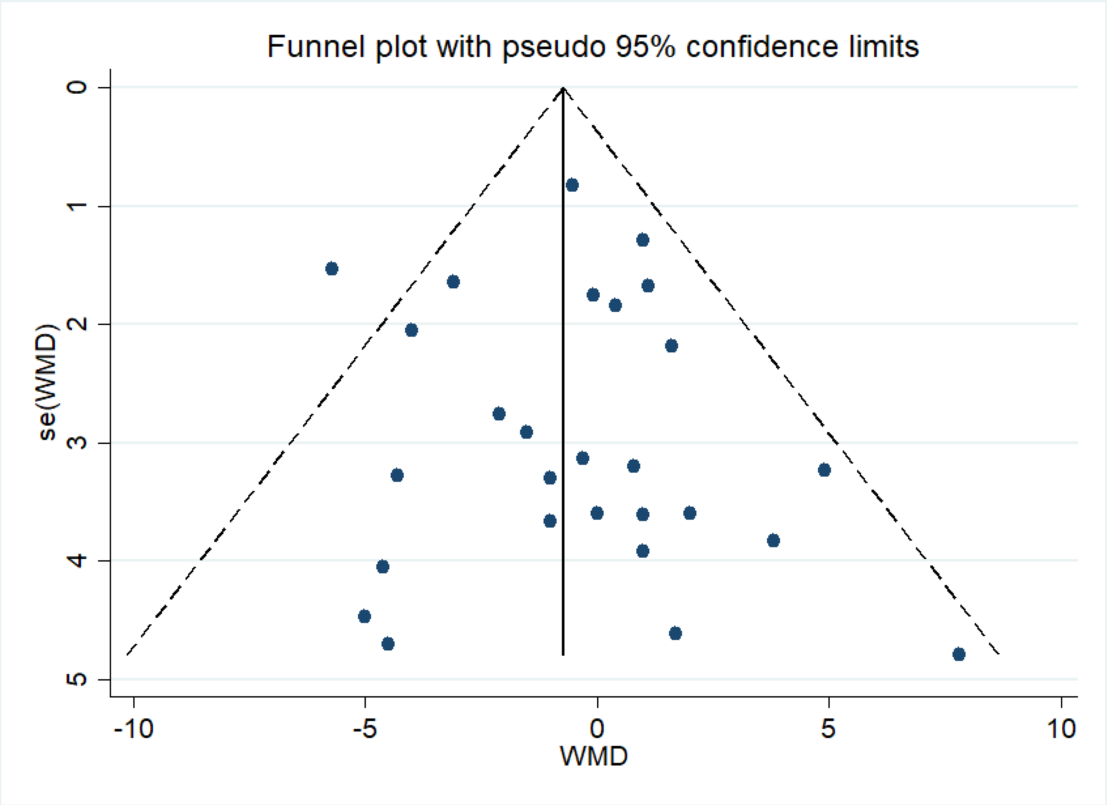

SUPPLEMENTARY FIGURE 16. Funnel plots for diastolic blood pressure

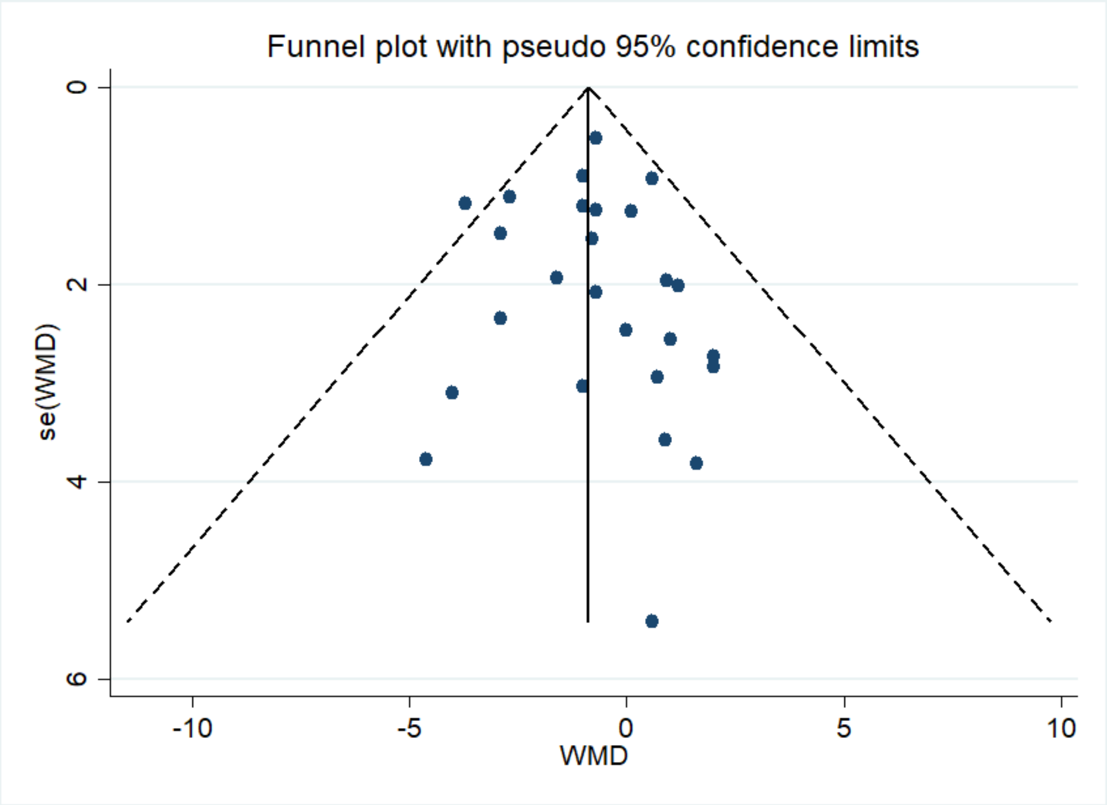

SUPPLEMENTARY FIGURE 17. Funnel plots for blood glucose

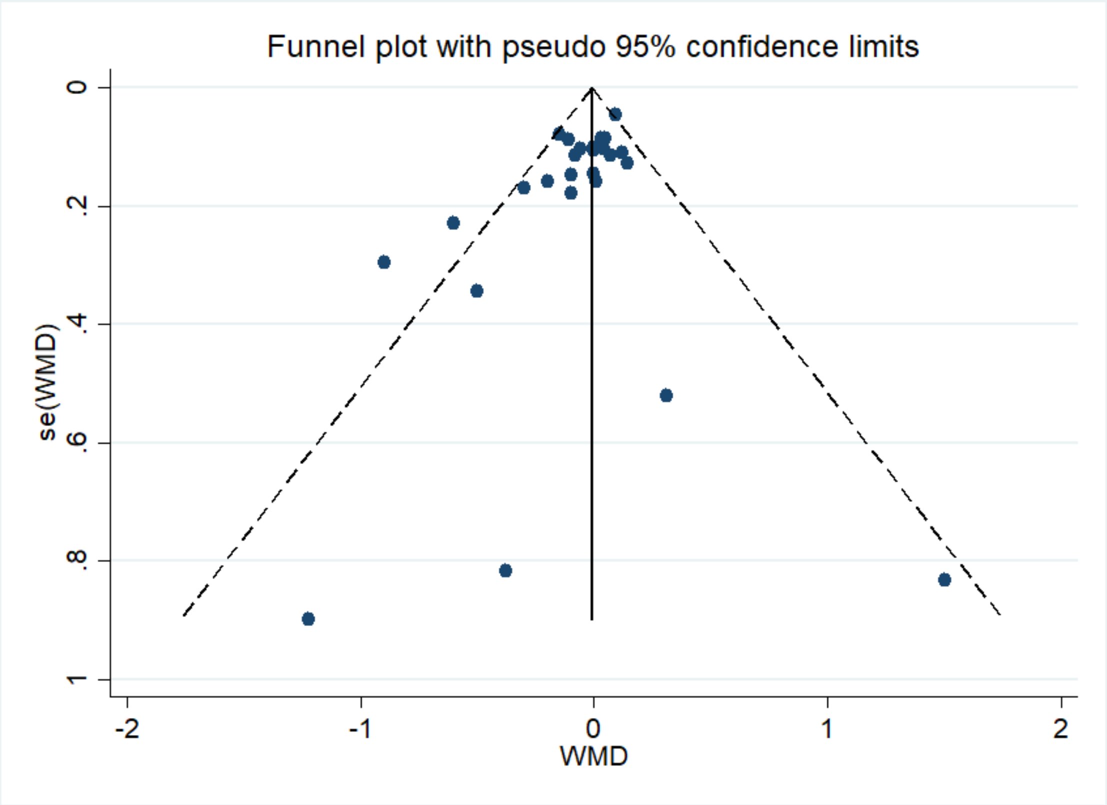

SUPPLEMENTARY FIGURE 18. Funnel plots for weight loss

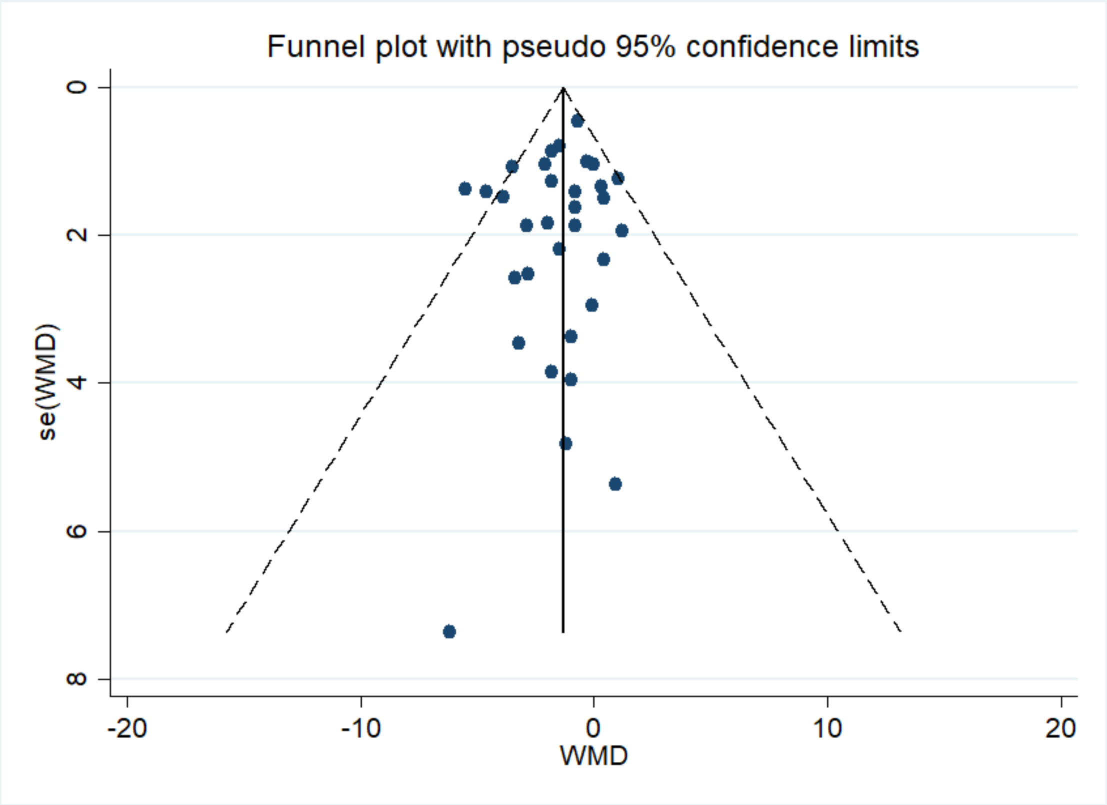

Supplement: Supplementary file 1 [file Data_Sheet_1.pdf]
